# Association of cardiometabolic factors and insulin resistance surrogates with mortality in participants from the Korean Genome and Epidemiology Study

**DOI:** 10.1186/s12944-023-01981-2

**Published:** 2023-12-01

**Authors:** Anthony Kityo, Sang-Ah Lee

**Affiliations:** 1 Department of Preventive Medicine, School of Medicine, Kangwon National University, Gangwon, Republic of Korea; 2 Interdisciplinary Graduate Program in Medical Bigdata Convergence, Kangwon National University, Gangwon, Republic of Korea

**Keywords:** Body mass index, Blood glucose, Triglycerides, All-cause mortality, Insulin resistance, Log-likelihood

## Abstract

**Background:**

Simple biochemical and anthropometric measurements such as fasting blood glucose (FBG), triglycerides (TG), high-density lipoprotein cholesterol (HDL-C), waist circumference (WC), and body mass index (BMI) are used to formulate insulin resistance (IR) indices. Whether these indices provide new predictive information for mortality remains unknown. This study examined the relationships of biochemical, anthropometric, and IR indices with mortality risk, as well as their predictive performance.

**Methods:**

The data source was the Korean Genome and Epidemiology Study (2004–2020) involving 114,957 participants whose data were linked to death records. The IR indices- triglyceride-glucose index (TyG), TyG-BMI, TyG-WC, visceral adiposity index (VAI), lipid accumulation product (LAP), and metabolic score for insulin resistance (METS-IR) were computed using standard formulae. The associations were examined using restricted cubic splines. The predictive performance was compared using the log-likelihood ratio chi-square test.

**Results:**

Body mass index was U-shaped, HDL-C was reverse J-shaped, and FBG and TG levels were J-shaped associated with all-cause mortality. Results showed U-shaped (TyG), J-shaped (TyG-BMI, VAI, LAP, and METS-IR), and reverse J-shaped (TyG-WC) associations with all-cause mortality. The percentages of new predictive information for all-cause mortality explained by the FBG level, BMI, TyG-BMI, and METIR were 3.34%, 2.33%, 1.47%, and 1.37%, respectively. Other IR indices and biochemical and anthropometric measurements provided < 1.0% of new predictive information. For cardiovascular disease mortality, the FBG, BMI, METIR, TyG-BMI, and HDL-C levels explained 2.57%, 2.12%, 1.59%, 1.30%, and 1.27% of new predictive information respectively. Moreover, the risks of cancer mortality explained by FBG level, VAI, and HDL-C level were 2.05%, 1.49%, and 1.28%, respectively.

**Conclusions:**

Fasting blood glucose level is a superior predictor of mortality risk and may be used as a simple predictive and preventative factor.

**Supplementary Information:**

The online version contains supplementary material available at 10.1186/s12944-023-01981-2.

## Background

Cardiovascular disease (CVD), cancer, and diabetes are among the major non-communicable diseases (NCDs) globally. Overweight/obesity and high blood glucose levels are among the three major metabolic risk factors for CVD and cancer [1]. Major chronic disease risk factors are linked to beta cell dysfunction, excessive insulin secretion, and impaired glucose tolerance, collectively referred to as insulin resistance (IR) [2, 3].

Insulin resistance primarily contributes to the development of various health conditions, such as cardiometabolic diseases [4–6], polycystic ovary syndrome [6], Alzheimer’s disease, chronic kidney disease (CKD) [7], and cancer [8]. This highlights the importance of early diagnosis of IR for the primary prevention of numerous NCDs. However, gold standard techniques for measuring insulin sensitivity are expensive, time-consuming, and may pose challenges in their implementation for epidemiological studies in certain settings [9].

Accordingly, simple biochemical and anthropometric measures are combined to compute the IR surrogate indices: a logarithmic product of fasting blood glucose (FBG) and triglycerides (TG) is used to define the triglyceride-glucose index (TyG) [10]; the metabolic score for insulin resistance (METS-IR) is computed from high density lipoprotein cholesterol (HDL-C), FBG, TG, and body mass index (BMI) [11]; lipid accumulation product (LAP) is derived from waist circumference (WC) and TG [12]; the visceral adiposity index (VAI) can be computed from TG, HDL-C, BMI and WC [13]; and products of TyG and BMI (TyG-BMI), or WC (TyG-WC) have also been suggested [14]. These indices have been evaluated for predicting both IR and vascular damage, and researchers have compared their predictive abilities [10, 11, 15–18]. However, few epidemiological studies have evaluated whether these indices are relevant in predicting mortality, and most of these studies have evaluated only the TyG index [19–23], while only one study has evaluated the VAI in relation to mortality [24]. Moreover, it is unknown whether these indices provide additional predictive information for mortality risk beyond the basic biochemical and anthropometric measurements from which they were computed. This study aimed to evaluate the association between six IR indices, alongside basic biochemical and anthropometric measurements, with mortality risk from all causes, cancer, and CVD. Additionally, the percentage of new predictive information for mortality provided by IR indices and basic measurements was compared. This study hypothesized that IR indices are associated with mortality, but do not provide additional predictive information for mortality beyond that provided by basic demographic, lifestyle, simple biochemical and anthropometric parameters. The focus of this study was non-insulin-based indices that can be conveniently computed from simple anthropometric and biochemical parameters, and are considered cost-effective and reliable markers of IR [10–18].

## Methods

This study followed the Strengthening the Reporting of Observational Studies in Epidemiology (STROBE) reporting guidelines.

### Study design and population

Individual participants were enrolled in the Korean Genome and Epidemiology Study-Health Examinees Cohort (KoGES-HEXA). Individuals (*n* = 173,195) from all eight regions of Korea were enlisted between 2004 and 2013. A total of 38 different health facilities served as recruitment centers [25, 26]. Among the 173,195 participants, 130,219 agreed to have their data connected to the National Statistical Office mortality records. After excluding 15,262 participants with missing IR index data, 114,957 participants (aged 40–79 years) were included in the analysis, of whom 75,251 (65.5%) were women (Fig. S1).

### Exposure variables

The main exposure variables were individual anthropometric (BMI and WC) and biochemical (FBG, HDL-C, and TG) parameters, and their composite IR indices (TyG, TyG-BMI, TyG-WC, METIR, LAP, and VAI), as described in the following subsections.

### Anthropometric measurements

The participants’ height (in meters) and weight (in kilograms) were recorded while they were barefoot and dressed in light clothing. For each individual, BMI was calculated as the fraction of their weight and the square of their height. WC was measured along the horizontal line equidistant from the lowest rib and the top of the hip bone.

### Biochemical measurements

After overnight fasting, a minimum of 19 cc of blood was collected in a serum separator tube and two tubes containing ethylenediaminetetraacetic acid. The samples were then transferred to conical tubes and subjected to further handling. Each biospecimen was assigned a unique identification that corresponded to each participant’s questionnaire and was marked with a 2D barcode sticker. Biospecimens were stored in refrigerators at each healthcare facility until they were transported by a courier from a commercial laboratory within 24 h. Subsequently, various tests were conducted on these specimens [25]. Baseline biochemical variables were quantified using enzymatic calorimetric methods with automatic analyzers (ADVIA 1650 and 1800; Siemens, Tarrytown, NY, USA). Surrogate IR indices were calculated based on anthropometric and biochemical measurements (Table 1).

**Table 1:** Calculation of IR indices

IR index	Formula
TyG [10]	ln (FBG [mg/dL] x triglyceride [mg/dL]/2)
TyG-BMI [14]	TyG x BMI
TyG-WC [14]	TyG x WC [cm]
METS-IR [11]	ln (2 × FBG [mg/dL] + triglyceride [mg/dL]) × BMI / ln (HDL-C[mg/dL])
VAI [13]	{WC [cm]/ (39.68 + (1.88 × BMI))) × triglycerides [mmol/L]/1.03 × (1.31/HDL-C [mmol/L]} in men, or {WC [cm]/ (36.58 + (1.89 × BMI))) × triglycerides [mmol/L]/0.81 × (1.51/ HDL-C [mmol]/L} in women
LAP [12]	(WC [cm] − 65) × triglycerides [mmol/L] in men, or (WC [cm] − 58) × triglycerides [mmol/L] in women

TyG, Triglyceride-glucose index; METS-IR, metabolic score for insulin resistance; VAI, visceral adiposity index; LAP, lipid accumulation product

### Outcome measures

Deaths from all causes, cancer, and CVD that occurred between recruitment and December 31, 2020 were ascertained by linking each participant to mortality records from the Korean National Statistical Office, or the National Health Insurance Service for Medicaid recipients. Cancer and CVD mortality was defined using the 10th revision of the International Classification of Disease codes (ICD 10).

### Assessment of covariates

A standardized interviewer-administered questionnaire was used to assess the age, sex, educational level, household income, region of residence, alcohol consumption, smoking habits, regular practice of physical exercise, and history of medical conditions. Covariates were defined as follows: Current alcohol drinking was defined as a history of alcohol consumption and consumption during study recruitment; current smoking was defined as a history of smoking more than 400 cigarettes over the participant’s lifetime, and current smoking during the study recruitment [27]; and regular physical exercise was defined as participating in activities that induced perspiration five or more days a week, with each session lasting for a minimum of 30 min.

The baseline prevalence of CVD, chronic obstructive pulmonary disease, chronic gastritis, and cancer were defined based on a self-reported diagnosis and intake of prescribed medication for the above conditions. A diagnosis of diabetes was based on FBG levels ≥ 126 mg/dl or being on diabetes treatment. Hypertension was determined based on the following clinical criteria: systolic blood pressure ≥ 130 mmHg, diastolic blood pressure ≤ 85 mmHg, or currently receiving antihypertensive pharmacotherapy [28]. The presence of CKD was based on an estimated glomerular filtration rate (eGFR) < 60 mL/min/1.73 m^2^ [29]. Disease scores were derived from the sum of the chronic morbidities at baseline.

### Statistical analysis

Person-years for each individual were computed by subtracting the study entry date from the date of death or December 31, 2020, with the earlier of the two considered first. Missing data on income was assigned “unknown” (8.7%), while missing data on each categorical covariate was replaced by the mode (< 5%). Continuous variables were expressed as either the mean value accompanied by its standard error, or the median value followed by its interquartile range. Categorical variables were expressed as frequencies (percentages). Participant characteristics were compared across fifths of TyG-BMI using general linear models and chi-square tests.

The participant characteristics associated with mortality in the Korean population were included in models as covariates [30]. The proportionality of hazards was evaluated using the Wald test. Nonlinear associations between basic biochemical and anthropometric measurements, IR indices, and mortality were modelled using restricted cubic spline (RCS) models [31]. Age (spline), sex, education level, monthly family income, region of residence, alcohol consumption, smoking habits, regular physical exercise, high sensitivity C-reactive protein (*hs*-CRP; continuous), disease score (only for all-cause mortality), baseline history of cancer (for cancer-specific mortality), and baseline history of CVD (for CVD-specific mortality) were included in the models.

Two steps were implemented to evaluate whether IR indices provided additional predictive information for mortality outcomes beyond basic anthropometric and biochemical measurements. First, a baseline model that included the covariates described above, was built for comparison with the model that additionally included individual measurements or their indices. Subsequently, the adequacy index (AI) was computed. The AI is the ratio of the log-likelihood (LL) of the baseline model to that of the models containing individual biochemical measurements or IR indices. The percentage of variation explained by each measurement (additional predictive information provided to the baseline model) was computed as (1-AI) x100. The percentage of variation indicates the differences in the risk of mortality due to a specific variable in the model [32].

As for sensitivity analyses, deaths reported in the first two years after study entry were excluded to account for latent period bias. Additionally, participants who reported at least one chronic disease at baseline were excluded.

The SAS statistical package (version 9.4; SAS Institute Inc., Cary, NC, USA) was used for data analyses. A *P* value < 0.05 was set as statistically significant.

## Results

The mean age (standard deviation) of the participants was 53.2 (8.3) years. Participants contributed 1,217,002 person-years (mean, 10.6 SE, 2.0) during which 3,567 deaths (cancer: 1,828; CVD: 599) were observed. Insulin resistance was positively correlated with old age, male sex, low educational level, low income, current smoking, high *hs*-CRP levels, and the presence of at least one chronic disease. However, IR was negatively correlated with current alcohol consumption and regular physical exercise (Table 2). The characteristics of participants were compared between the original cohort and the analytical sample. No substantial differences in the distribution of demographic, lifestyle, and clinical characteristics were reported between the entire cohort and the analytical sample (Table S1).

**Table 2:** Description of study participants

Characteristic	Fifths of TyG-BMI index (*N* = 114,957)				
	Q1	Q2	Q3	Q4	Q5
	22,991	22,991	22,993	22,991	22,991
Age, years, mean (SE)	50.9 (0.1)	52.5 (0.1)	53.6 (0.1)	54.4 (0.1)	54.4 (0.1)
BMI, kg/m^2^, mean (SE)	20.5 (0.0)	22.5 (0.0)	23.7 (0.0)	25.1 (0.0)	27.7 (0.0)
Male, n (%)	4,207 (18.3)	5,104 (22.2)	5,909 (25.7)	6,644 (28.9)	6,782 (29.5)
Elementary education, n (%)	2,028 (8.8)	2,656 (11.6)	3,312 (14.4)	4,215 (18.3)	5,053 (22.0)
Income, < 1000USD, n (%)	1,748 (7.6)	1,859 (8.1)	2,154 (9.4)	2,537 (11.0)	2,913 (12.7)
Single/others, n (%)	2,527 (11.0)	2,180 (9.5)	2,274 (9.9)	2,354 (10.2)	2,726 (11.9)
Rural residents, n (%)	7,195 (31.3)	7,146 (31.1)	7,115 (30.9)	7,379 (32.1)	7,764 (33.8)
Current smokers, n (%)	3,196 (13.9)	3,487 (15.2)	3,555 (15.5)	3,612 (15.7)	3,514 (15.3)
Current drinkers, n (%)	10,587 (46.1)	10,534 (45.8)	10,503 (45.7)	10,028 (43.6)	10,101 (43.9)
Regular exercise, n (%)	7,984 (34.7)	8,472 (36.9)	8,727 (38.0)	8,433 (36.7)	7,634 (33.2)
Elevated *hs*-CRP, n (%)	1,071 (4.7)	1,176 (5.1)	1,418 (6.2)	1,621 (7.1)	2,454 (10.7)
History of Cancer, n (%)	854 (3.7)	731 (3.2)	766 (3.3)	752 (3.3)	712 (3.1)
History of CVD, n (%)	328 (1.4)	443 (1.9)	555 (2.4)	651 (2.8)	750 (3.3)
Disease score, ≥ 1, n (%)	1,372 (6.0)	1,691 (7.4)	2,005 (8.7)	2,134 (9.3)	2,495 (10.9)
TyG, median (IQR)	7.9 (7.6–8.2)	8.3 (7.9–8.5)	8.5 (8.2–8.8)	8.7 (8.4-9.0)	9.1 (8.7–9.4)
TyG-BMI, median (IQR)	164 (164 − 155)	184 (178–191)	199 (193–208)	218 (210–224)	247 (236–261)
TyG-WC, median (IQR)	572 (533–620)	634 (593–685)	678 (636–731)	727 (684–777)	808 (755–865)
METS-IR, median (IQR)	27.0 (25.9–29.4)	31.3 (29.7–33.2)	34.0 (32.4–36.0)	37.1 (35.4–39.2)	42.6 (40.0-45.6)
VAI, median (IQR)	1.8 (1.3–2.3)	2.2 (1.6–2.9)	2.6 (1.9–3.4)	3.0 (2.3–3.9)	3.8 (2.8–5.2)
LAP, median (IQR)	8.5 (5.6–12.3)	15.9 (11.7–21.5)	23.2 (17.4–31.2)	33.4 (25.2–44.6)	54.5 (39.9–77.2)

SE, standard error; IQR, interquartile range; BMI, body mass index; TyG, triglyceride-glucose index; WC, waist circumference; VAI, visceral adiposity index; LAP, lipid accumulation product; METS-IR, metabolic score for insulin resistance; CVD, cardiovascular disease; *hs*-CRP, high sensitivity C-reactive protein

**Fig. 1:**
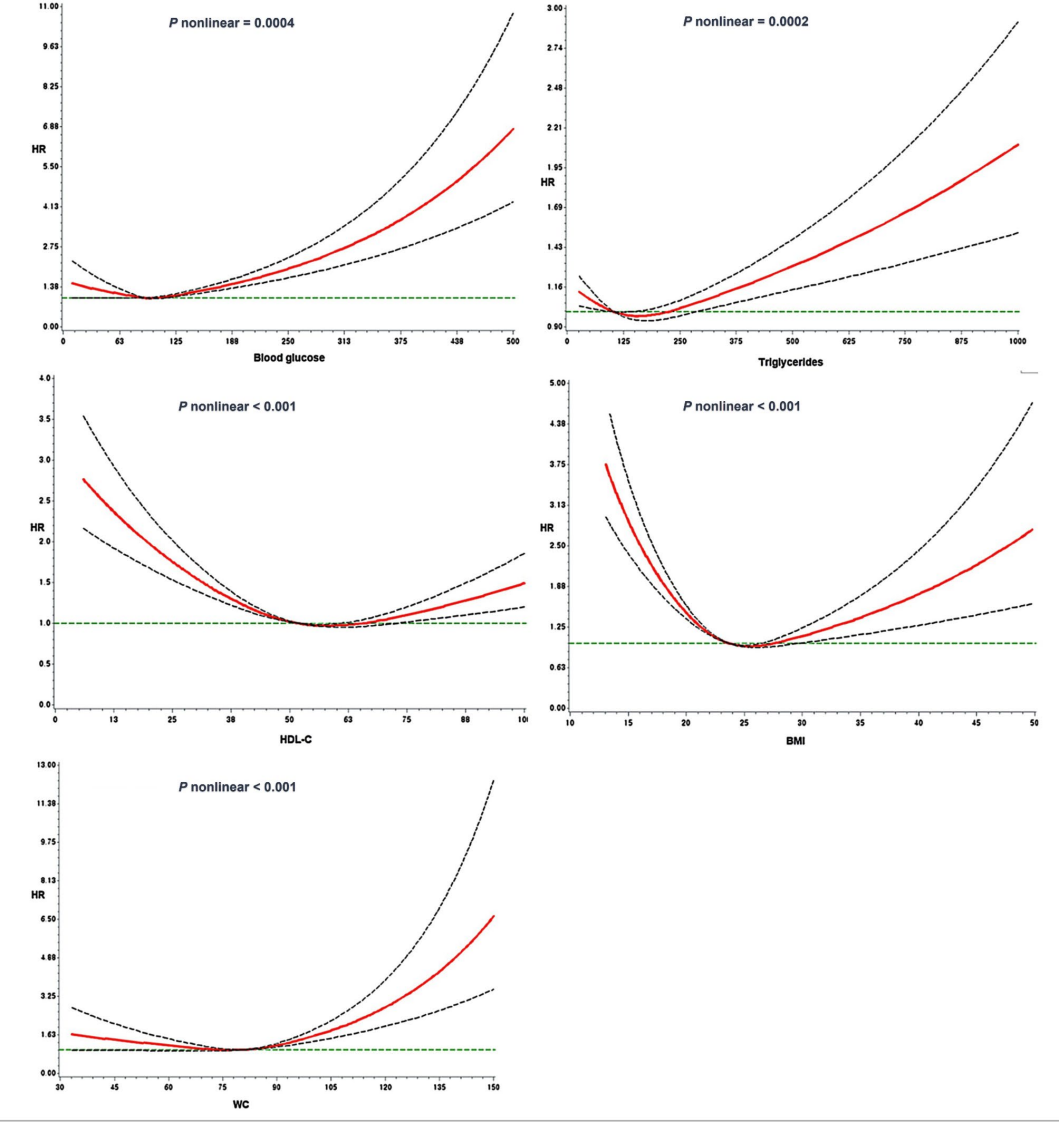
Nonlinear association of plasma biomarkers and anthropometric measures with all-cause mortality. The models were fitted using RCS with knots located at the 5th, 50th, and 95th percentiles of the individual measurements, and were djusted for age, sex, education, monthly family income, marital status, smoking, drinking, regular physical exercise, and disease scores. The solid line are hazard ratios (HR), and the dotted lines are corresponding 95% confidence intervals (CI). BMI, body mass index; WC, waist circumference; HDL-C, high-density lipoprotein cholesterol

Simple biochemical and anthropometric measurements were nonlinearly associated with all-cause mortality (*P* < 0.0001) (Fig. 1). FBG and TG levels were J-shaped, HDL-C was reverse J-shaped, and BMI was U-shaped associated with all-cause mortality. These results persisted after excluding participants who died two years after recruitment (Fig. S2), and those with chronic diseases at baseline (Fig. S3).

FBG levels were linearly associated with a high risk of death from cancer (Fig. S4) and CVD (Fig. S5). However, J-shaped associations with cancer mortality were observed for the other biochemical and anthropometric measurements (Fig. S4). Moreover, increased HDL-C levels were associated with a linear decrease; but BMI and WC were J-shaped associated with CVD mortality (Fig. S5).

**Fig. 2:**
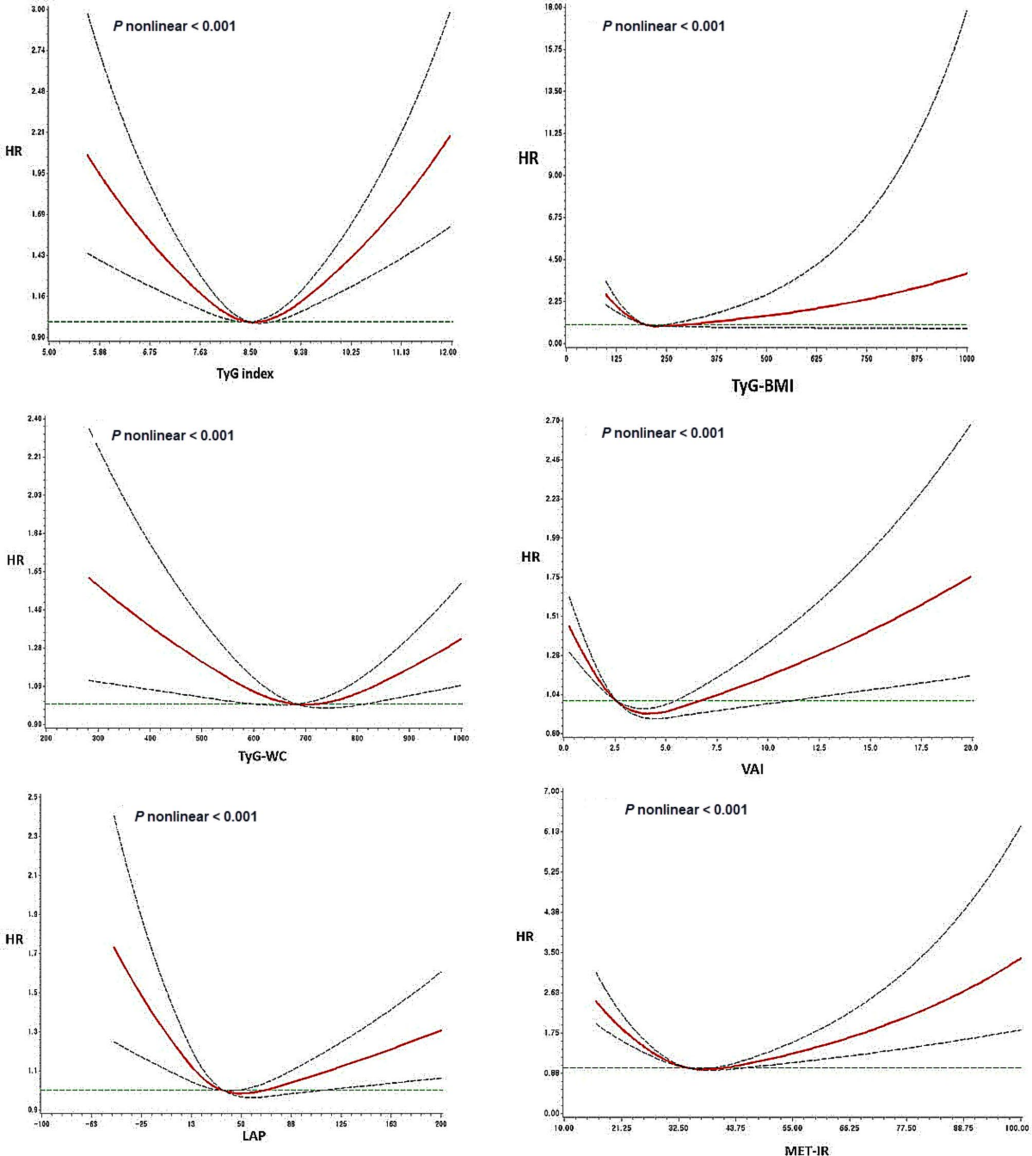
Nonlinear association of IR indices with all-cause mortality. The models were fitted using RCS with knots positioned at the 5th, 50th, and 95th percentiles of the individual measurements, and were adjusted for age, sex, education, monthly family income, marital status, smoking, drinking, regular physical exercise, and disease scores. The solid line are hazard ratios (HR), and the dotted lines are corresponding 95% confidence intervals (CI). TyG, triglyceride-glucose index; VAI, visceral adiposity index; LAP, lipid accumulation product; METS-IR, metabolic score for insulin resistance.

Associations between IR indices and all-cause mortality were significantly nonlinear (*P* < 0.0001), with a U-shaped relationship observed with TyG, a J-shaped association with TyG-BMI, VAI, LAP, and METS-IR, and a reverse J-shaped association with TyG-WC (Fig. 2). These associations persisted in the sensitivity analyses (Fig. S6 and S7).

A high risk of cancer mortality associated with lower levels of IR indices was reported, except for the TyG index (Fig. S9). High TyG and TyG-WC were linearly associated with an increased risk of CVD mortality. However, METIR and TyG-BMI were J-shaped in relation to CVD mortality (Fig. S10).

FBG was the most highly ranked predictor of mortality outcomes compared to other biochemical markers and IR indices, explaining the highest variation (new predictive information) in all-cause (3.34%), CVD (2.6%), and cancer mortality (2.05%), followed by BMI (all-cause mortality, 2.33%; CVD mortality, 2.12%) and HDL-C (1.28% for cancer mortality). However, among the IR indices, TyG-BMI contributed 1.50% of the predictive information for all-cause mortality, followed by METIR (1.37%). In addition, METIR and VAI contributed to the highest percentages of variation in CVD-(1.59%) and cancer-specific (1.49%) mortality, respectively (Table 3).

**Table 3:** Percentage of variation in mortality outcomes explained by basic anthropometric and biochemical markers, and IR indices

Model	All-cause mortality			CVD mortality			Cancer mortality	
	AI^a^	% new information^b^		AI	% new information		AI	% new information
Baseline^c^	1.00	-		1.00	-		1.00	-
+Triglycerides	0.993	0.741		0.998	0.184		0.993	0.716
+Glucose	0.967	3.337		0.974	2.570		0.979	2.052
+HDL-C	0.990	1.044		0.987	1.268		0.987	1.285
+BMI	0.977	2.329		0.979	2.120		0.995	0.537
+WC	0.993	0.713		0.993	0.698		0.998	0.248
+TyG	0.993	0.739		0.992	0.822		0.997	0.273
+TyG-BMI	0.985	1.467		0.987	1.290		0.997	0.345
+TyG-WC	0.994	0.640		0.994	0.629		0.997	0.275
+METIR	0.986	1.369		0.984	1.586		0.998	0.237
+VAI	0.990	0.956		0.999	0.147		0.985	1.496
+LAP	0.991	0.911		0.999	0.148		0.994	0.605

BMI, body mass index; WC, waist circumference; HDL-C, high density lipoprotein cholesterol; TyG, triglyceride-glucose index; VAI, visceral adiposity index; LAP, lipid accumulation product; METS-IR, metabolic score for insulin resistance; AI, adequacy index;

^a^Log-likelihood _baseline model_/ Log-likelihood _model including biomarker/IR index_

^b^(1-AI)*100

^c^Included age (spline), sex, education, monthly family income, marital status, smoking, drinking, regular physical exercise, disease score and *hs-*CRP. The baseline models for CVD and cancer mortality additionally included baseline history of CVD and cancer respectively

## Discussion

### Study findings

Various IR indices have been formulated based on basic physical and biochemical measurements obtained from routine clinical examinations. However, whether these composite IR indices provide additional predictive information for mortality beyond the basic measurements is unknown. This large cohort study evaluated the nonlinear associations of IR indices and basic biochemical and anthropometric measurements with mortality, and compared their predictive performance. Basic measurements and composite indices were both nonlinearly associated with all-cause mortality, and FBG level was a superior predictor of all-cause, cancer, and CVD mortality. These results suggest that FBG, rather than composite IR indices, may be a convenient and less expensive approach for screening individuals at risk of future premature mortality and targeting them for lifestyle and pharmacological interventions.

### Comparison with previous studies

The discrimination between IR and glycemic control has been evaluated using different IR surrogates in recent studies. The TyG index superseded the Homeostatic Model Assessment of Insulin Resistance (HOMA-IR), VAI, and LAP in predicting diabetes [33]. In a study with middle-aged Chinese individuals, TyG and LAP were better predictors of IR than TG/HDL-C ratio and VAI [15]. The product of TyG and obesity indices demonstrated the best discrimination of IR measured using HOMA-IR [16]. Notably, these studies employed the area under the receiver operating characteristic curve (AUROC), which is a less sensitive tool for assessing predictive performance [32]. To the best of our knowledge, no studies have compared mortality prediction by IR surrogate indices using more robust measures of predictive performance. In the current study, FBG levels explained the highest variation in total, cancer-, and CVD-specific mortality compared to the IR indices, suggesting that additional processing of basic measurements to compute composite indices does not add predictive information to mortality models beyond basic measurements.

The relationship between IR indices and mortality risk has been previously explored. High TyG index levels were associated with a significant risk of all-cause mortality [19–23], with two studies reporting a U-shaped association [21, 22]. High LAP levels are associated with a high risk of mortality [14], but an inverse relationship per unit increment has been reported in men [34]. In patients with high CVD risk, LAP (instead of BMI) was positively associated with high mortality risk [35]. Furthermore, LAP significantly predicted mortality risk among postmenopausal women of normal weight [36]. Notably, a few large cohort studies have reported an association between LAP and mortality, but none of these studies evaluated nonlinear associations. In the first large cohort in the United Kingdom, the total mortality risk was high among individuals with high VAI values [2]. Two studies reported an increased mortality risk in patients with kidney disease and a high VAI [37, 38], with a J-shaped association found in one study [37]. Both the lowest and highest METS-IR values are related to total and CVD-mortality among individuals with hyperglycemia [39]; however, no large-scale studies have been conducted in generally healthy individuals.

### Possible explanations of study results

The nonlinear associations between the IR indices and mortality observed in our study align with those of previous reports, specifically for TyG [21, 22], VAI [37], and METS-IR [39]. A potential explanation for this observation is that most endocrine hormones exhibit a range of physiological concentrations, and the functions of an organism are compromised below or above the physiological range [40]. Some researchers have hypothesized that IR in the absence of glucose dysmetabolism can increase longevity in certain populations such as individuals with obesity. Individuals with obesity with the lowest HOMA-IR values have the lowest survival rates and highest risk of CVD-related mortality [41]. Therefore, considering the potential benefits of IR in individuals with obesity, it can be inferred that the absence of IR in these individuals may deactivate the necessary internal self-defense mechanisms against obesity [41]. Moreover, the pharmacological reduction of IR in humans may result in organ damage [42], and the removal of IR defense mechanisms has been implicated in an increased CVD risk [43].

### Study strengths and limitations

The major strength of our study is the comprehensive comparison of multiple biochemical, anthropometric, and surrogate markers of IR with mortality in a large cohort with a relatively long follow-up period. A substantial number of participants and a long follow-up duration enabled the accrual of adequate events and allowed the evaluation of cause-specific mortality. The use of objectively measured anthropometric and biochemical variables to compute IR indices and accurately ascertain mortality through vital records reduced measurement error and increased the validity of our findings. Moreover, the prospective study design reduced the possibility of reverse causation and strengthened the causal conclusions. In addition, the study accounted for multiple covariates, which potentially minimized confounding factors. Finally, the LL ratio, used to compare model performance, is considered the gold standard for evaluating and measuring the new information provided by a biomarker to a minimal model [32]. In contrast, we used anthropometric and biochemical measurements that were measured once at baseline, which precluded accounting for long-term changes. Nevertheless, single anthropometric and biochemical measurements have been shown to approximate average values, owing to their stability over time [43]. Although we included multiple covariates in our models, there is a possibility of residual unmeasured confounding.

## Conclusions

Routinely measured biochemical and anthropometric parameters and composite indices of IR demonstrated nonlinear associations with all-cause mortality. An increase in FBG was associated with an increase in the risk of CVD mortality; however, a linear increase and decrease in cancer-related mortality were observed for FBG and HDL-C, respectively. The FBG level was the most informative predictor of all-cause, CVD-, and cancer-specific mortality. This study showed that surrogate measures of insulin resistance do not provide additional predictive information for mortality beyond FBG levels. Therefore, FBG levels can be utilized as a simple and less expensive predictive and preventative marker of the future risk of premature mortality, and should be routinely monitored in the general population.

### Electronic supplementary material

Below is the link to the electronic supplementary material.

**Supplementary Material 1**: Flow chart showing participant selection, and restricted cubic spline curves for sensitivity analyses and cause-specific mortality

## Data Availability

Data from the Health Examinees (HEXA) study is part of the Korean Genome and Epidemiology Study (KoGES), conducted by Korea Disease Control and Prevention Agency (KDCA). The dataset analyzed in this study is maintained and managed by the Division of Population Health Research at the National Institute of Health, Korean Disease Control and Prevention Agency. It contains personal data that may potentially be sensitive to the patients, even though researchers are provided with an anonymized dataset that excludes resident registration numbers. Accordingly, the minimal data set used in the current study could not be publicly shared by the authors due to legal restriction on sharing sensitive patient information. To access the data, researchers are required to submit ethics approval, and a detailed research plan to the KDCA. Upon approval, the researchers are required to physically visit the KCDA and conduct the analysis from the KoGES data analysis room at the KCDA in Osong, Chungcheong Province, Republic of Korea. However, if the analysis does not involve linkage to the cancer registry, virtual access to the anonymized data set can be granted. Other researchers may request access to the anonymized data by contacting the following individuals at the Division of Population Health Research, National Institute of Health, Korea Disease Control and Prevention Agency: Senior Staff Scientist Dr. Jung Hyun Lee (jaylee1485@korea.kr); Director Dr. Kyoungho Lee (khlee3789@korea.kr).

## List of abbreviations

AI: Adequacy index
BMI: Body mass index
CKD: Chronic kidney disease
CVD: Cardiovascular disease
eGFR: Estimated glomerular filtration rate
FBG: Fasting blood glucose
HEXA: Health Examinees Study
HDL-C: High density lipoprotein cholesterol
*hs*CRP: High sensitivity C-reactive protein
ICD: International Classification of Disease
IR: Insulin resistance
KoGES: Korean Genome and Epidemiology Study
LAP: Lipid accumulation product
LL: Log likelihood
METIR: Metabolic score for insulin resistance
NCD: Non-communicable disease
RCS: Restricted cubic splines
TG: Triglyceride
TyG: Triglyceride-glucose index
VAI: Visceral Adipose Index
WC: Waist circumference

## References

[1] WHO. Noncommunicable diseases [Internet]. Accessed 2023 Nov 3. Available from: https://www.who.int/data/gho/data/themes/noncommunicable-diseases.

[2] Esser N, Utzschneider KM, Kahn SE (2020). Early beta cell dysfunction vs insulin hypersecretion as the primary event in the pathogenesis of dysglycaemia. Diabetologia.

[3] Johnson JD (2021). On the causal relationships between hyperinsulinaemia, insulin resistance, obesity and dysglycaemia in type 2 Diabetes. Diabetologia.

[4] Wondmkun YT, Obesity (2020). Insulin resistance, and type 2 Diabetes: associations and therapeutic implications. Diabetes Metab Syndr Obes.

[5] Ormazabal V, Nair S, Elfeky O, Aguayo C, Salomon C, Zuñiga FA (2018). Association between insulin resistance and the development of Cardiovascular Disease. Cardiovasc Diabetol.

[6] Diamanti-Kandarakis E, Dunaif A (2012). Insulin resistance and the polycystic ovary syndrome revisited: an update on mechanisms and implications. Endocr Rev.

[7] Li M, Chi X, Wang Y, Setrerrahmane S, Xie W, Xu H (2022). Trends in insulin resistance: insights into mechanisms and therapeutic strategy. Sig Transduct Target Ther.

[8] Inoue M, Tsugane S (2012). Insulin resistance and cancer: epidemiological evidence. Endocr Relat Cancer.

[9] DeFronzo RA, Tobin JD, Andres R (1979). Glucose clamp technique: a method for quantifying insulin secretion and resistance. Am J Physiol.

[10] Simental-Mendía LE, Rodríguez-Morán M, Guerrero-Romero F (2008). The product of fasting glucose and triglycerides as surrogate for identifying insulin resistance in apparently healthy subjects. Metab Syndr Relat Disord.

[11] Bello-Chavolla OY, Almeda-Valdes P, Gomez-Velasco D, Viveros-Ruiz T, Cruz-Bautista I, Romo-Romo A (2018). METS-IR, a novel score to evaluate insulin sensitivity, is predictive of visceral adiposity and incident type 2 Diabetes. Eur J Endocrinol.

[12] Taverna MJ, Martínez-Larrad MT, Frechtel GD, Serrano-Ríos M (2011). Lipid accumulation product: a powerful marker of metabolic syndrome in healthy population. Eur J Endocrinol.

[13] Kahn HS, Valdez R (2003). Metabolic risks identified by the combination of enlarged waist and elevated triacylglycerol concentration. Am J Clin Nutr.

[14] Er LK, Wu S, Chou HH, Hsu LA, Teng MS, Sun YC (2016). Triglyceride glucose-body Mass Index is a simple and clinically useful surrogate marker for insulin resistance in nondiabetic individuals. PLoS ONE.

[15] Huang R, Cheng Z, Jin X, Yu X, Yu J, Guo Y (2022). Usefulness of four surrogate indexes of insulin resistance in middle-aged population in Hefei, China. Ann Med.

[16] Lee J, Kim B, Kim W, Ahn C, Choi HY, Kim JG (2021). Lipid indices as simple and clinically useful surrogate markers for insulin resistance in the U.S. population. Sci Rep.

[17] Lim J, Kim J, Koo SH, Kwon GC (2019). Comparison of triglyceride glucose index, and related parameters to predict insulin resistance in Korean adults: an analysis of the 2007–2010 Korean National Health and Nutrition Examination Survey. PLoS ONE.

[18] Fiorentino TV, Marini MA, Succurro E, Andreozzi F, Sesti G (2019). Relationships of surrogate indexes of insulin resistance with insulin sensitivity assessed by euglycemic hyperinsulinemic clamp and subclinical vascular damage. BMJ Open Diabetes Res Care.

[19] Muhammad IF, Bao X, Nilsson PM, Zaigham S (2022). Triglyceride-glucose (TyG) index is a predictor of arterial stiffness, incidence of Diabetes, Cardiovascular Disease, and all-cause and cardiovascular mortality: a longitudinal two-cohort analysis. Front Cardiovasc Med.

[20] Zhou D, Liu X-C, Kenneth L, Huang Y-Q, Feng Y-Q (2022). A Non-linear Association of triglyceride Glycemic Index with Cardiovascular and all-cause Mortality among patients with Hypertension. Front Cardiovasc Med.

[21] Liu XC, He GD, Lo K, Huang YQ, Feng YQ (2021). The triglyceride-glucose index, an insulin resistance marker, was non-linear Associated with all-cause and Cardiovascular Mortality in the General Population. Front Cardiovasc Med.

[22] Sun M, Guo H, Wang Y, Ma D (2022). Association of triglyceride glucose index with all-cause and cause-specific mortality among middle age and elderly US population. BMC Geriatr.

[23] Kim KS, Hong S, Hwang YC, Ahn HY, Park CY (2022). Evaluating triglyceride and glucose index as a simple and easy-to-calculate marker for all-cause and Cardiovascular Mortality. J Gen Intern Med.

[24] He Q, Liu S, Feng Z, Li T, Chu J, Hu W (2022). Association between the visceral adiposity index and risks of all-cause and cause-specific mortalities in a large cohort: findings from the UK biobank. Nutr Metab Cardiovasc Dis.

[25] Kang D, Kim DH, Kim DH, Lee DH, Lee DH, Lee HJ (2015). The Health examinees (HEXA) study: Rationale, Study Design and Baseline characteristics. Asian Pac J Cancer Prev.

[26] Kim Y, Han B-G (2017). The KoGES group. Cohort Profile: the Korean Genome and Epidemiology Study (KoGES) Consortium. Int J Epidemiol.

[27] Yang JJ, Song M, Yoon H-S, Lee H-W, Lee Y, Lee S-A (2015). What are the major determinants in the success of Smoking Cessation: results from the Health examinees Study. PLoS ONE.

[28] Alberti KGMM, Zimmet P, Shaw J (2006). Metabolic syndrome - a new world-wide definition. A consensus statement from the International Diabetes Federation. Diabet Med.

[29] Levey AS, Stevens LA, Schmid CH, Zhang Y, Castro AF, Feldman HI (2017). Predictors of all-cause mortality among 514,866 participants from the Korean National Health Screening Cohort. PLoS ONE.

[30] Ahn C, Hwang Y, Park SK (2017). A new equation to estimate glomerular filtration rate. Ann Intern Med.

[31] Desquilbet L, Mariotti F (2010). Dose-response analyses using restricted cubic spline functions in public health research. Stat Med.

[32] Harrell FE (2015). Regression modeling strategies.

[33] Kim B, Choi HY, Kim W, Ahn C, Lee J, Kim JG (2018). The cut-off values of surrogate measures for insulin resistance in the Korean population according to the Korean Genome and Epidemiology Study (KOGES). PLoS ONE.

[34] Bozorgmanesh M, Hadaegh F, Azizi F (2010). Predictive performances of lipid accumulation product vs. adiposity measures for Cardiovascular Diseases and all-cause mortality, 8.6-year follow-up: Tehran lipid and glucose study. Lipids Health Dis.

[35] Ioachimescu A, Brennan D, Hoar B, Hoogwerf B (2010). The lipid accumulation product and all-cause mortality in patients at high cardiovascular risk: a PreCIS database study. Obes (Silver Spring).

[36] Wehr E, Pilz S, Boehm B, Marz W, Obermayer-Pietsch B (2011). The lipid accumulation product is associated with increased mortality in normal weight postmenopausal women. Obes (Silver Spring).

[37] Yan LJ, Zeng YR, Chan-Shan Ma RN, Zheng Y (2022). J-shaped association between the visceral adiposity index and all-cause mortality in patients with chronic Kidney Disease. Nutrition.

[38] Chen HY, Chiu YL, Chuang YF, Hsu SP, Pai MF, Yang JY (2014). Visceral adiposity index and risks of cardiovascular events and mortality in prevalent hemodialysis patients. Cardiovasc Diabetol.

[39] Wang Z, Xie J, Wang J, Feng W, Liu N, Liu Y (2022). Association between a novel metabolic score for insulin resistance and mortality in people with Diabetes. Front Cardiovasc Med.

[40] Kolb H, Kempf K, Röhling M, Martin S, Insulin (2020). Too much of a good thing is bad. BMC Med.

[41] Kim KS, Lee YM, Lee IK, Kim DJ, Jacobs DR, Lee DH (2015). Paradoxical associations of insulin resistance with Total and Cardiovascular Mortality in humans. J Gerontol A Biol Sci Med Sci.

[42] Barzilai N, Ferrucci L (2012). Insulin resistance and aging: a cause or a protective response?. J Gerontol A Biol Sci Med Sci.

[43] Jackson SE, van Jaarsveld CH, Beeken RJ (2015). Four-year stability of anthropometric and cardio-metabolic parameters in a prospective cohort of older adults. Biomark Med.

