# Supplementary material for: Association of cardiometabolic factors and insulin resistance surrogates with mortality in participants from the Korean Genome and Epidemiology Study
Source: Lipids Health Dis. 2023 Dec 1;22:210. doi: 10.1186/s12944-023-01981-2 (PMC10691157; doi:10.1186/s12944-023-01981-2)
Supplement: Supplementary file 1 — Supplementary Material 1: Flow chart showing participant selection, and restricted cubic spline curves for sensitivity analyses and cause-specific mortality [file 12944_2023_1981_MOESM1_ESM.docx]

**Supplementary Material**

**Association of Cardiometabolic Factors and Insulin Resistance Surrogates with Mortality in Participants from the Korean Genome and Epidemiology Study**

Anthony Kityo, Sang-Ah Lee


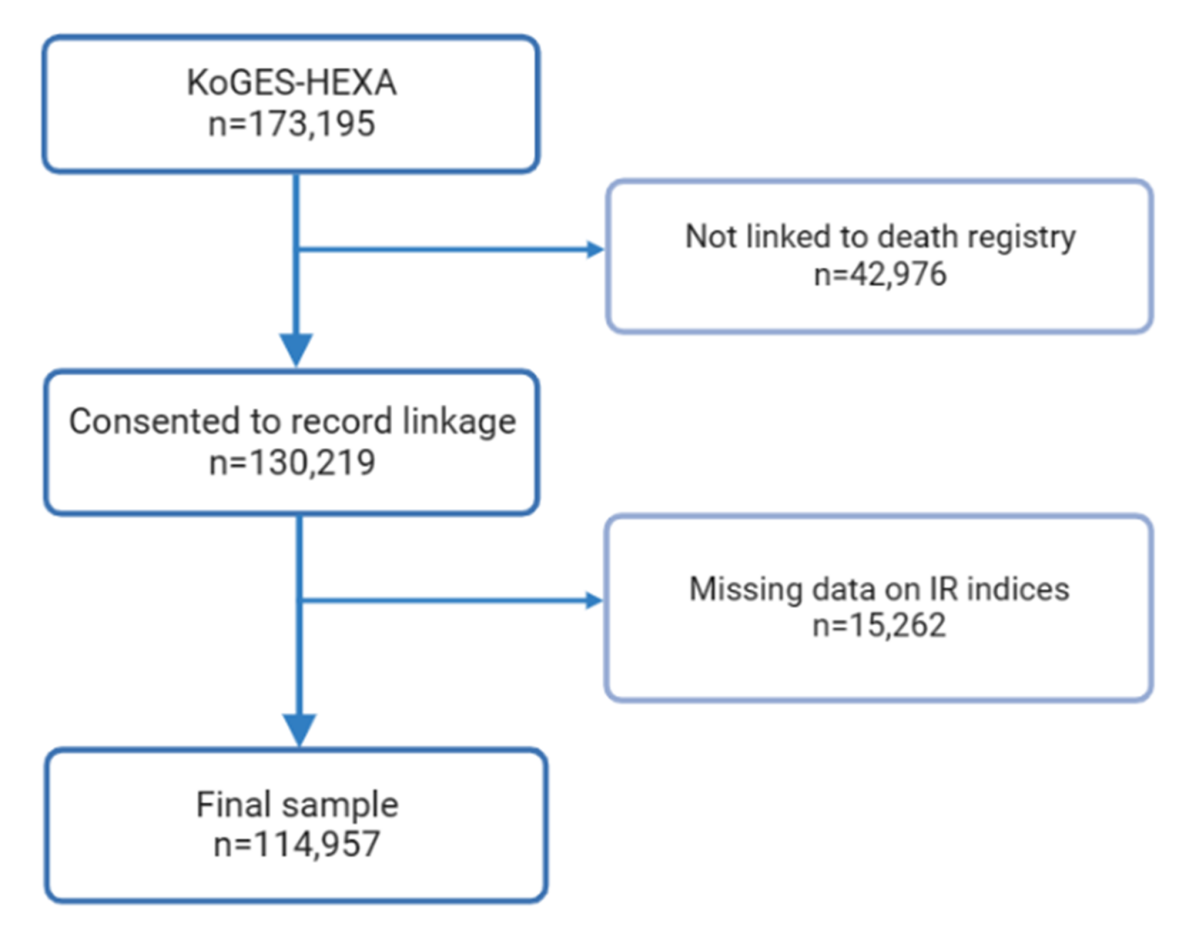


Fig.S1. Flowchart illustrating study participant selection

| 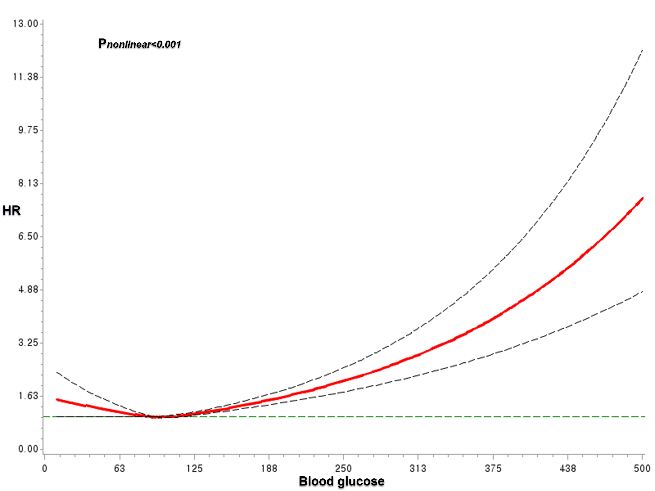 | 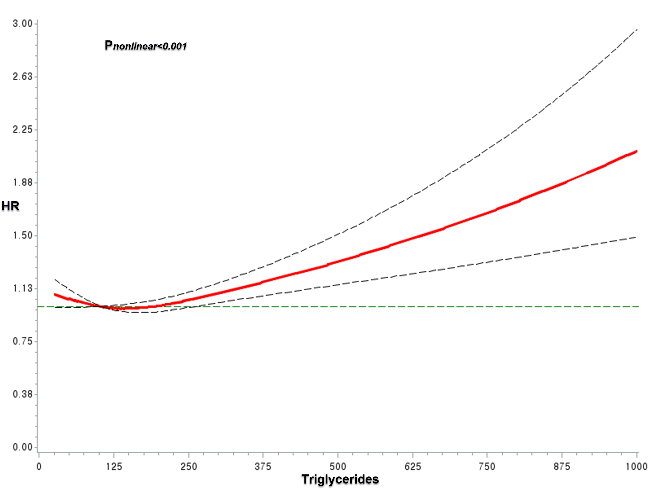 |
| --- | --- |
| 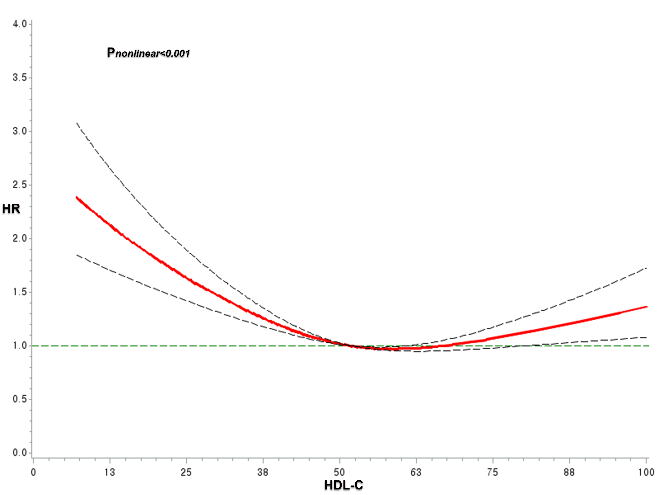 | 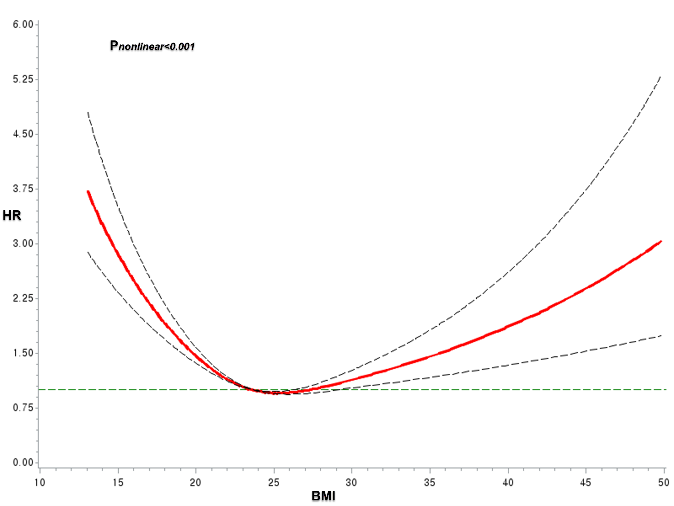 |
| 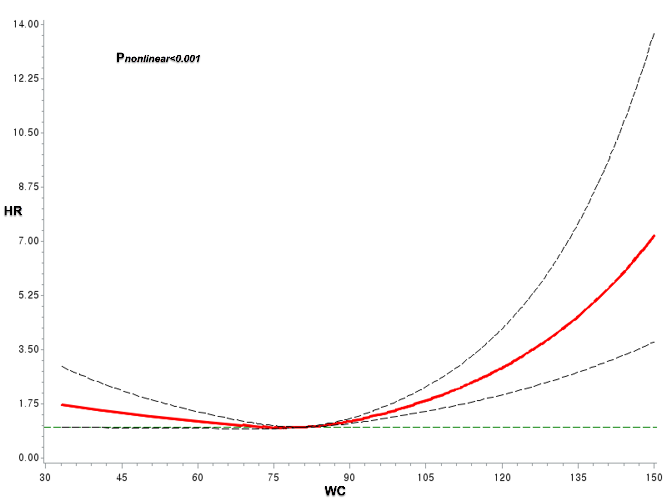 |  |

Fig.S2. **Nonlinear association between individual biomarkers and all-cause mortality after excluding participants who died in the first 2 years of follow up. Events/total, 3,447/114,637.** The model was fitted using regression splines with three knots at the 5th, 50th, and 95th percentiles of IR indices. The red line indicates hazard ratios (HR), and the dotted lines indicate the 95% CI. The models were adjusted for age, sex, education, monthly family income, marital status, smoking, drinking, regular physical exercise, and disease scores. BMI, body mass index; WC, waist circumference; HDL-C, high-density lipoprotein cholesterol.

| 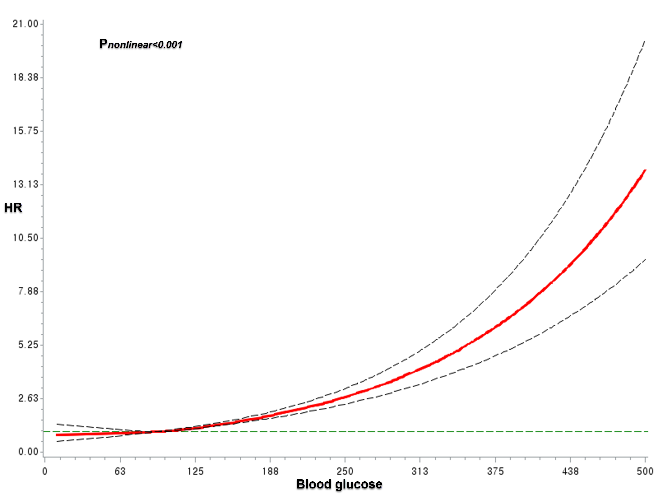 | 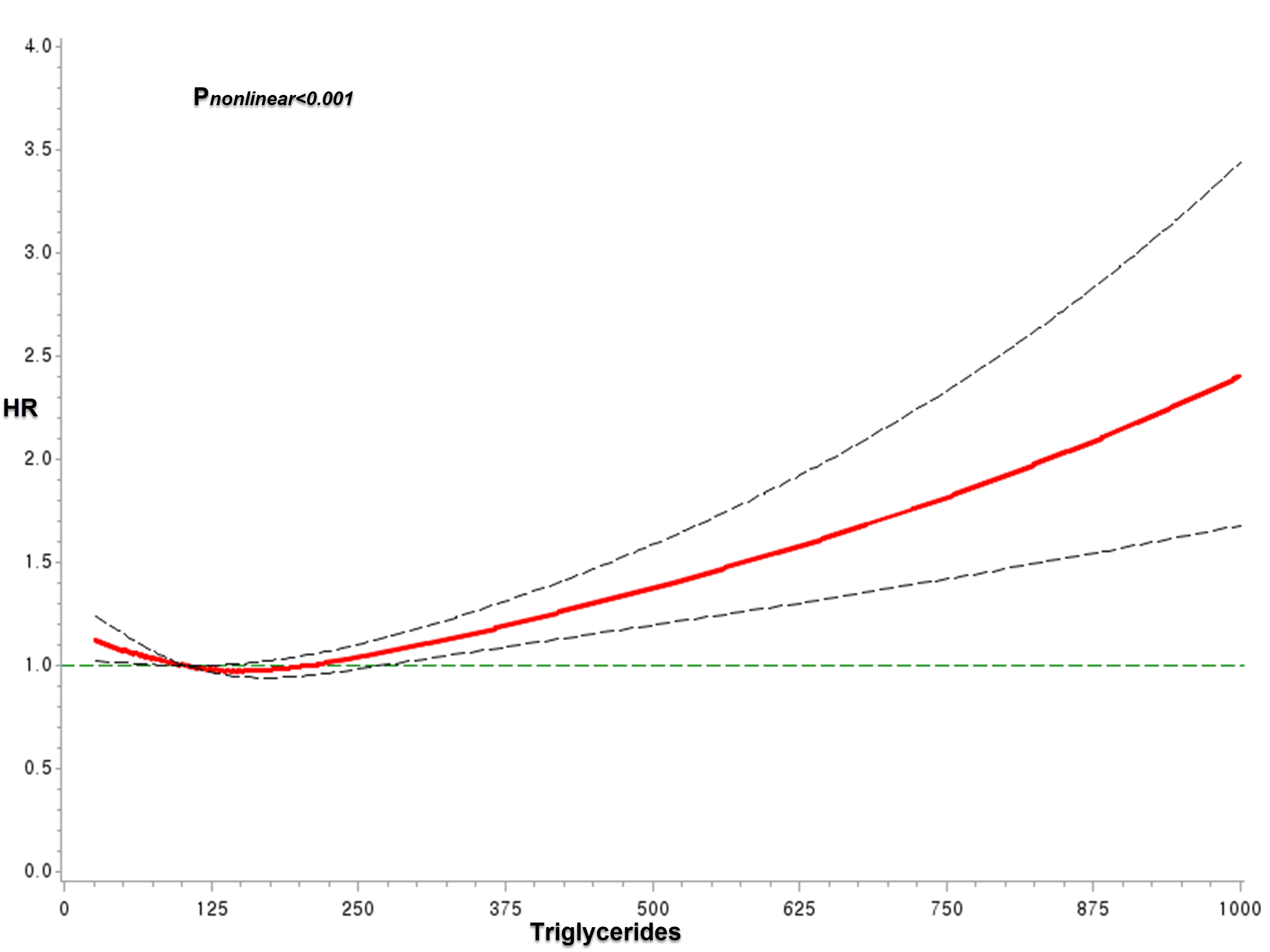 |
| --- | --- |
| 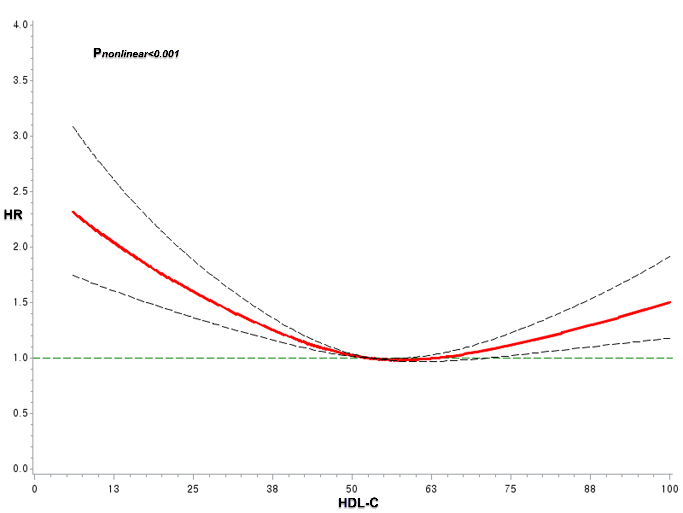 | 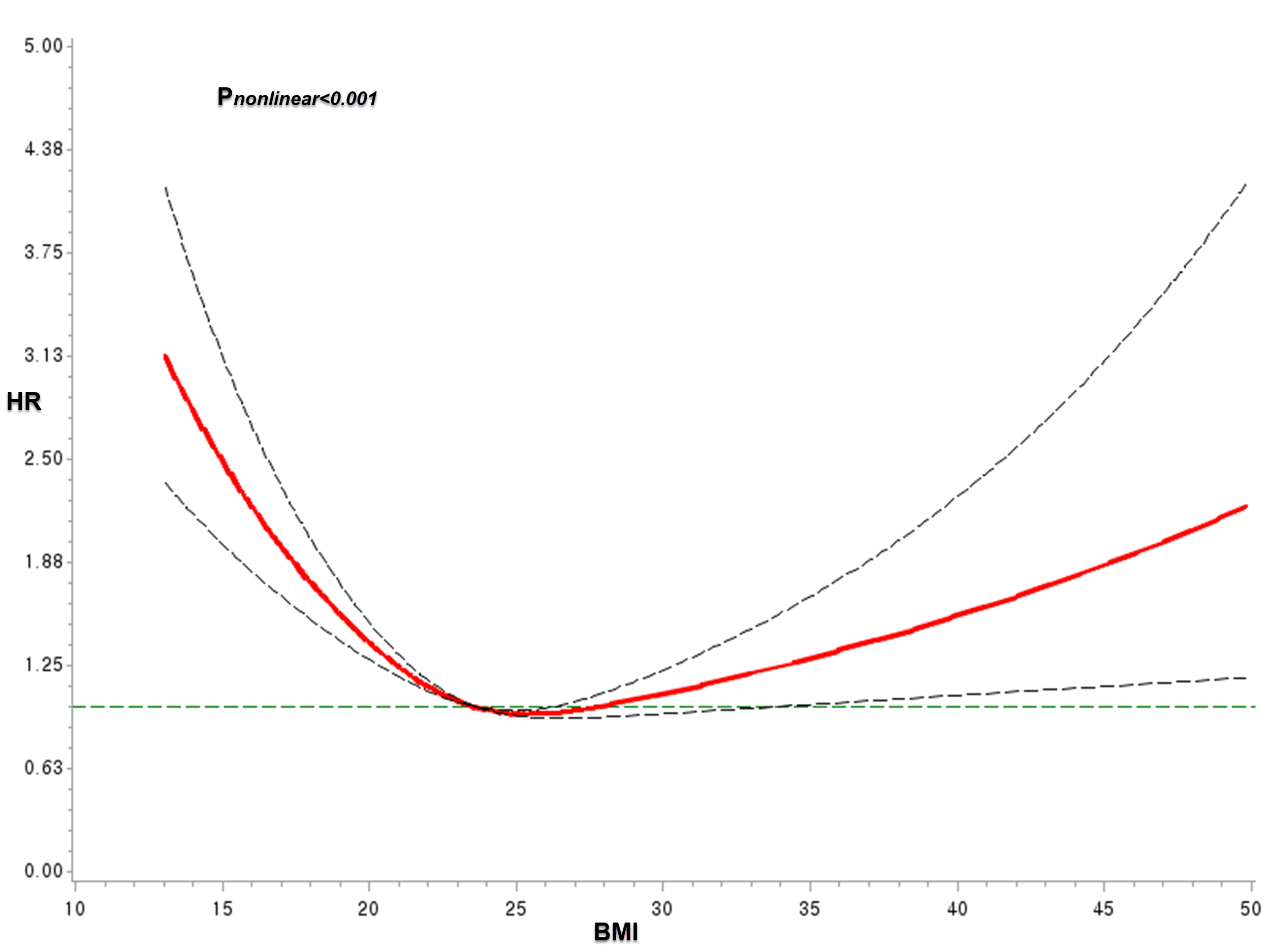 |
| 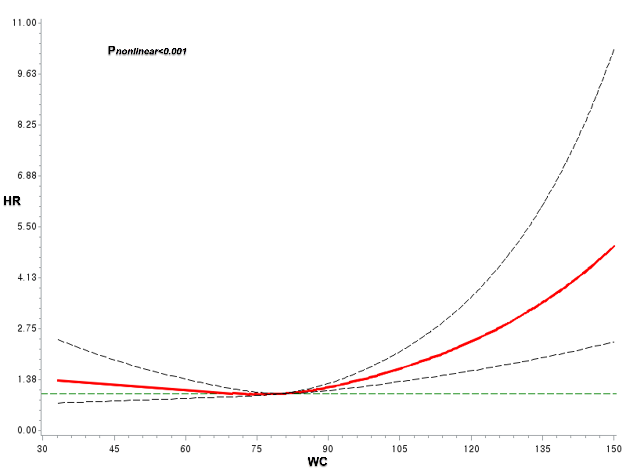 |  |

Fig.S3 **Nonlinear association between individual biomarkers and all-cause mortality after excluding participants with a disease score>0 at baseline. Events/total, 2,917/105,260.** The model was fitted using regression splines with three knots at the 5th, 50th, and 95th percentiles of IR indices. The red line indicates hazard ratios (HR), and the dotted lines indicate the 95% CI. The models were adjusted for age, sex, education, monthly family income, marital status, smoking, drinking, regular physical exercise, and disease scores. BMI, body mass index; WC, waist circumference; HDL-C, high-density lipoprotein cholesterol.

| 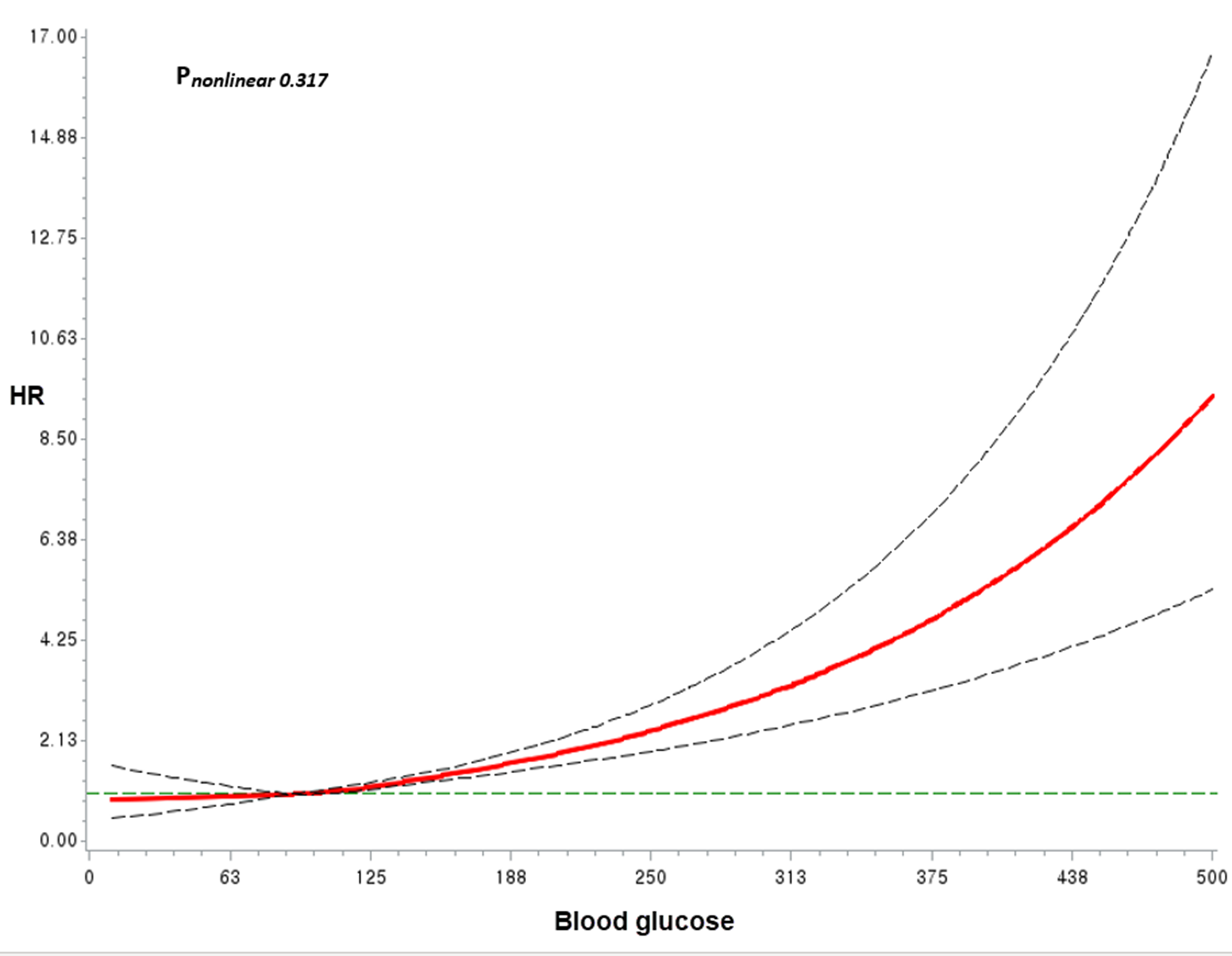 | 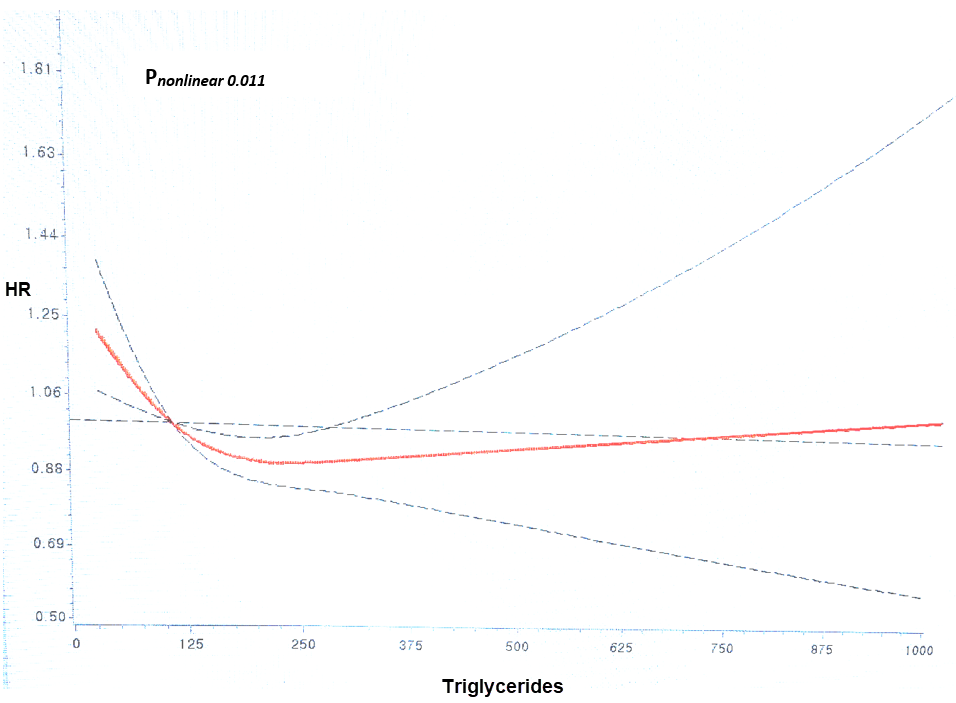 |
| --- | --- |
| 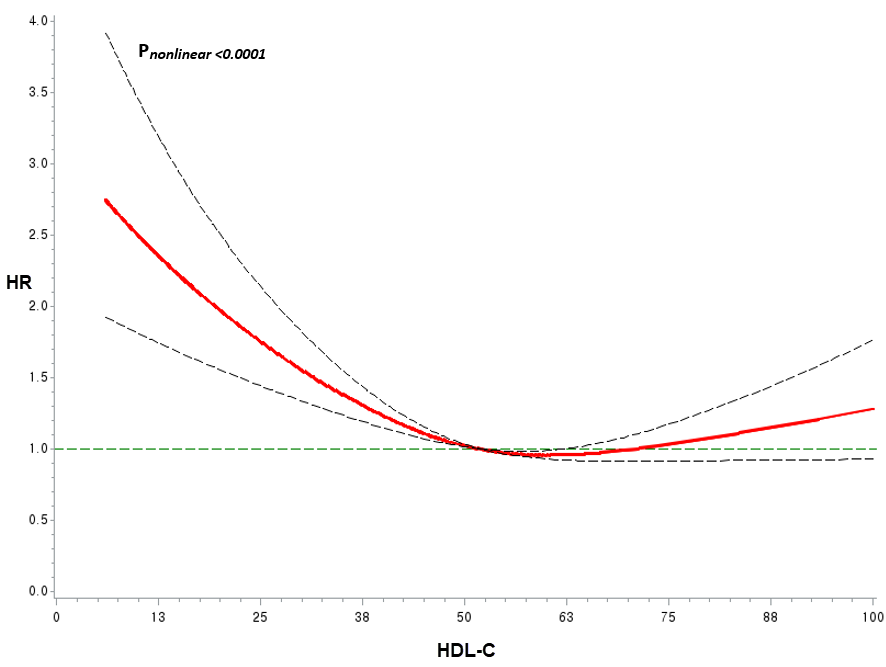 | 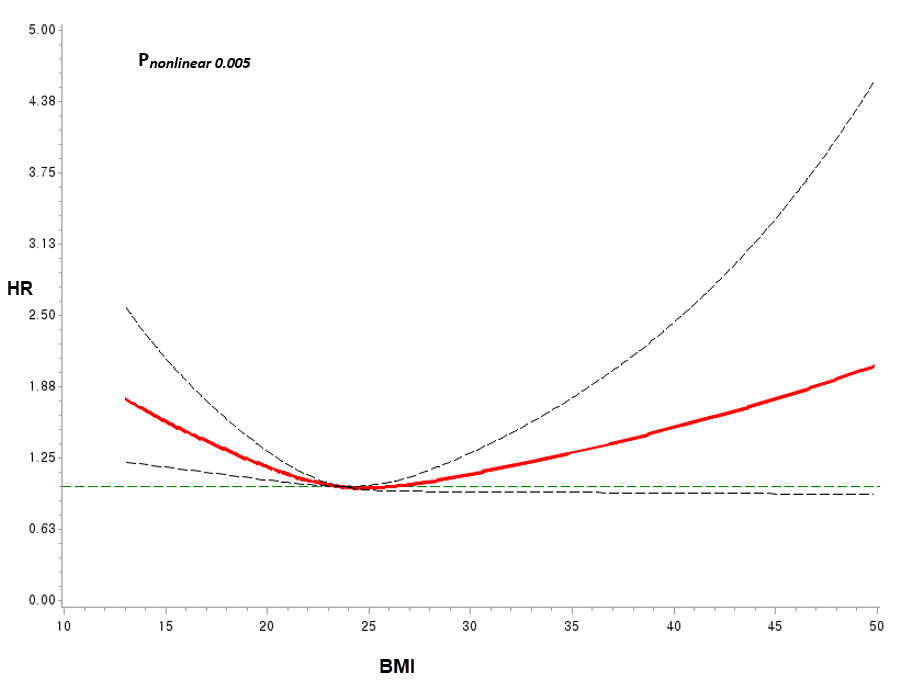 |
| 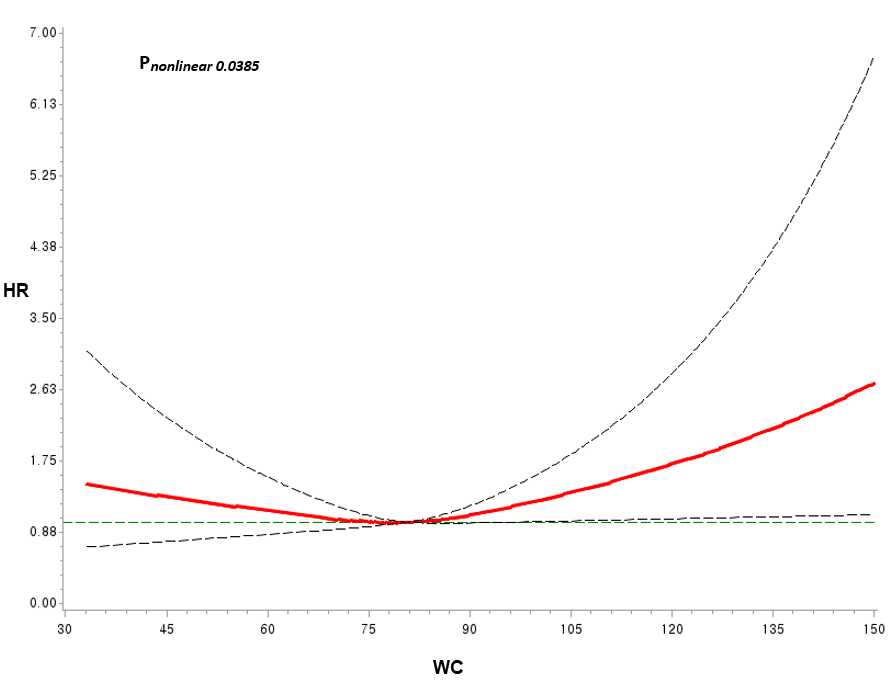 |  |

Fig.S4. Nonlinear association between basic measurements and cancer mortality. The model was fitted using regression splines with three knots at the 5th, 50th, and 95th percentiles of IR indices. The red line indicates hazard ratios (HR), and the dotted lines indicate the 95% CI. The model was adjusted for age, sex, educational level, monthly income, marital status, smoking, drinking, regular physical exercise, and BMI, and history of cancer. BMI, body mass index.

| 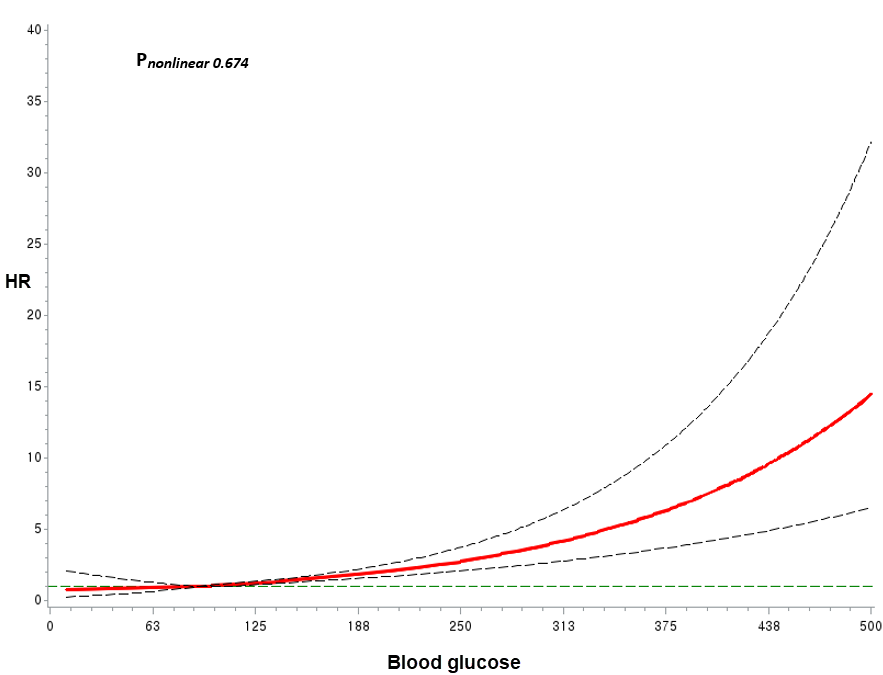 | 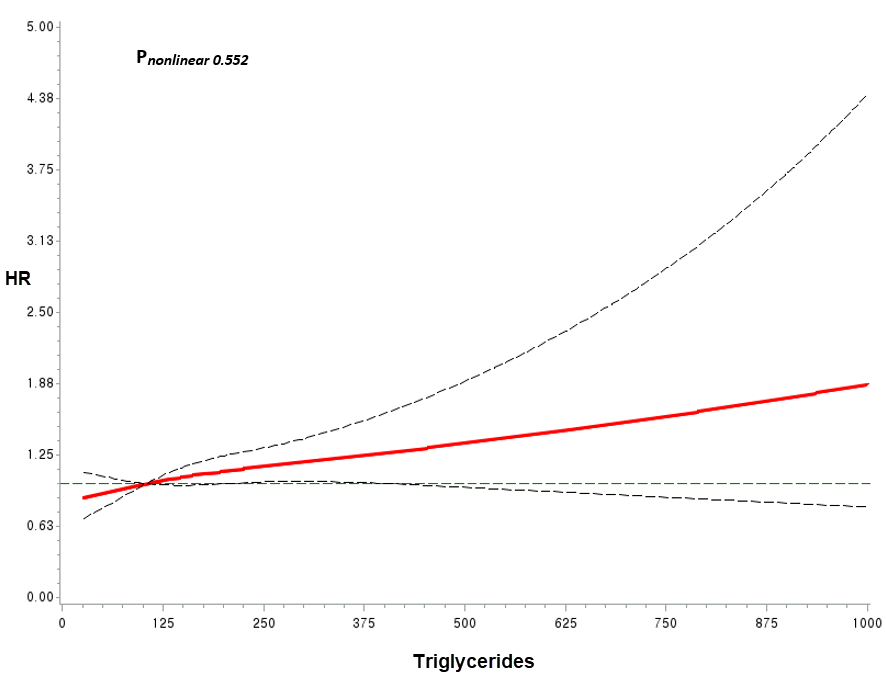 |
| --- | --- |
| 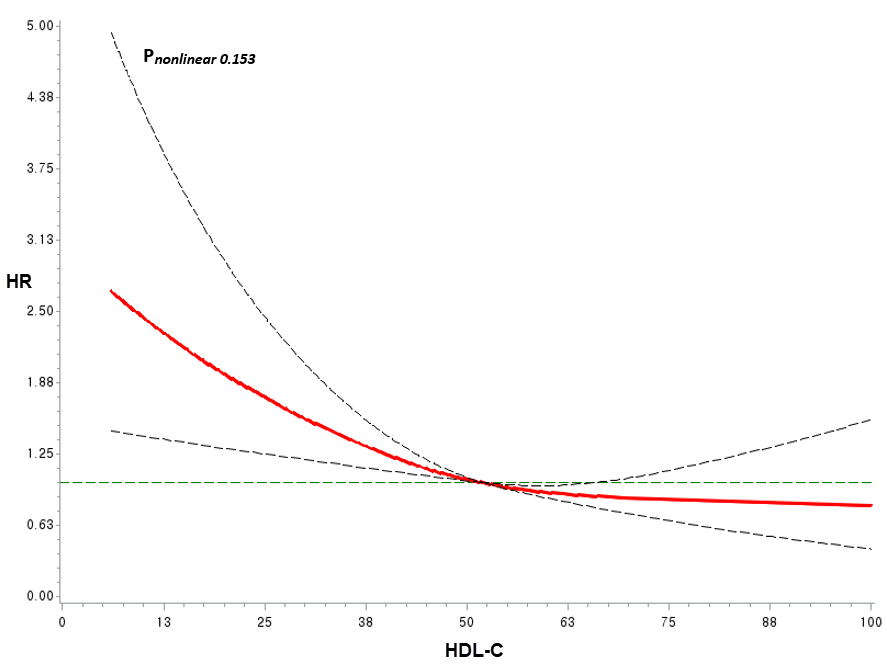 | 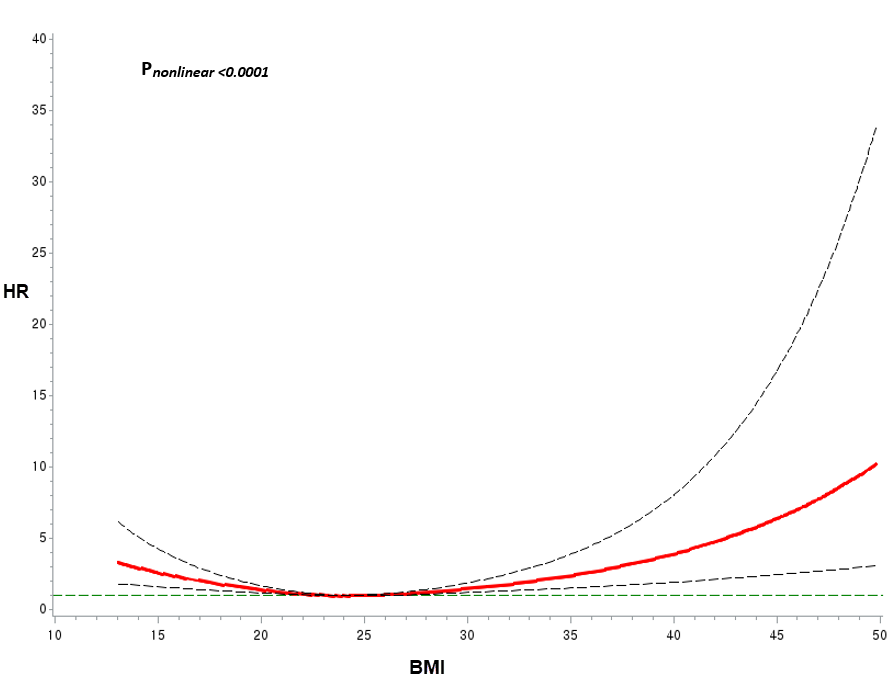 |
| 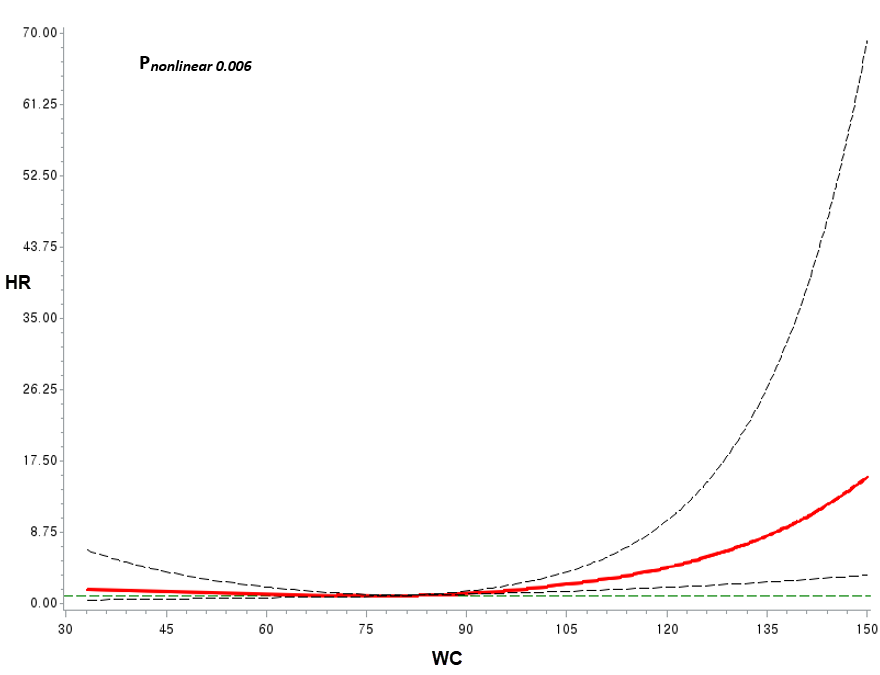 |  |

Fig.S5. Nonlinear association between basic measurements and CVD mortality. The model was fitted using regression splines with three knots at the 5th, 50th, and 95th percentiles of IR indices. The red line indicates hazard ratios (HR), and the dotted lines indicate the 95% CI. The model was adjusted for age, sex, educational level, monthly income, marital status, smoking, drinking, regular physical exercise, BMI, and history of cancer. BMI, body mass index; WC, waist circumference; HDL-C, high density lipoprotein cholesterol.

| 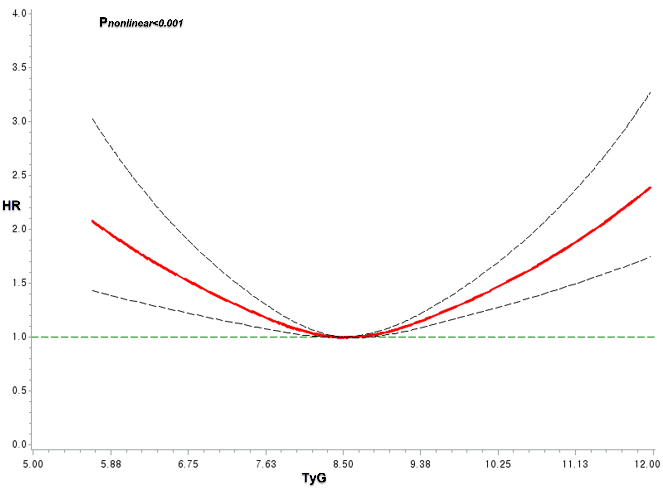 | 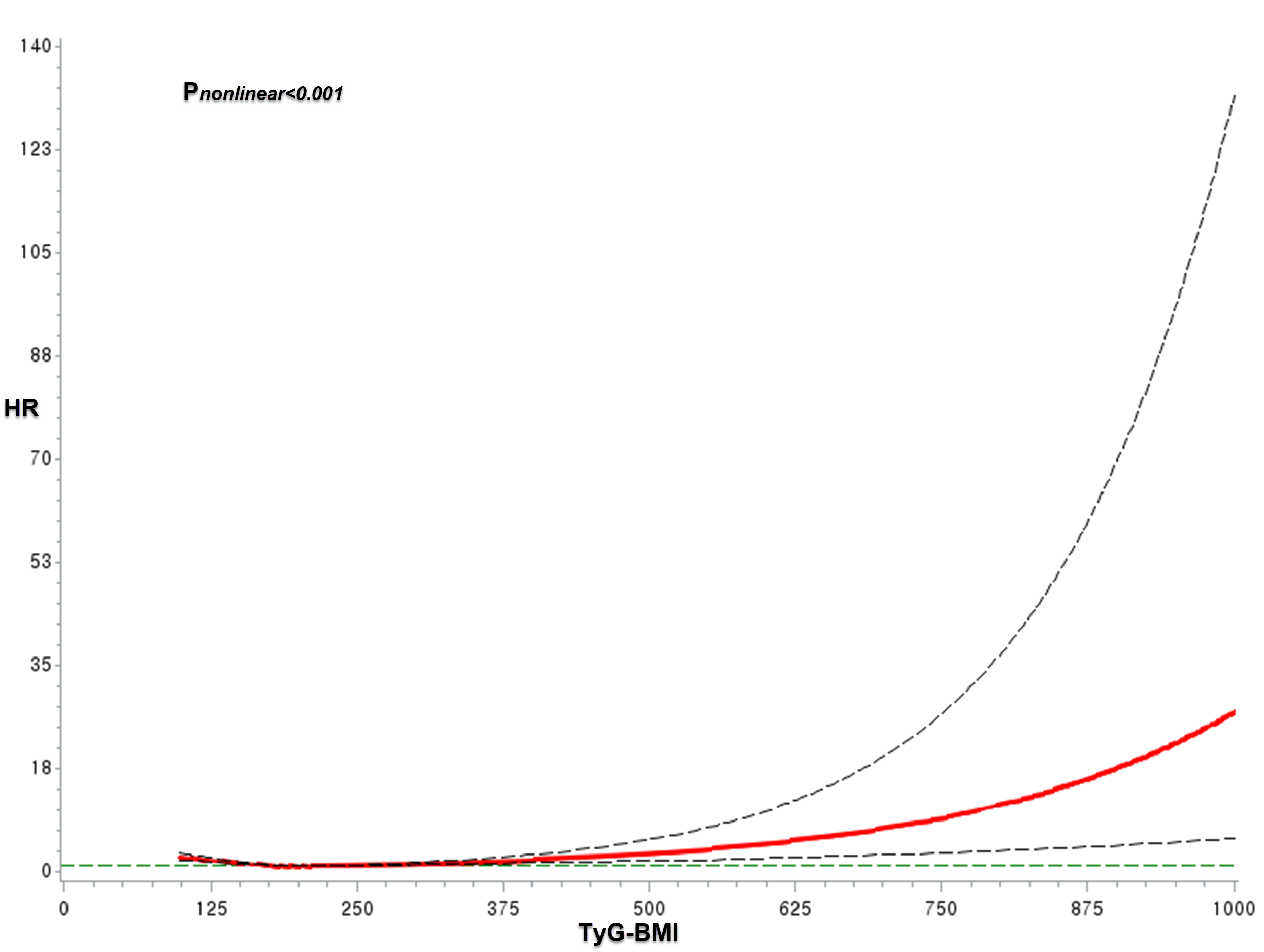 |
| --- | --- |
| 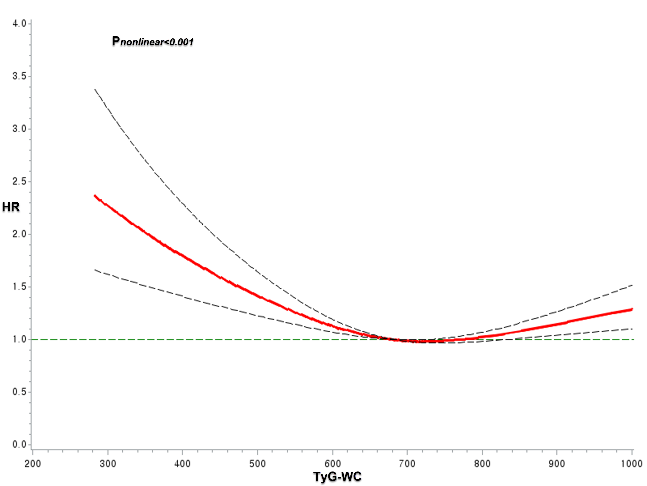 | 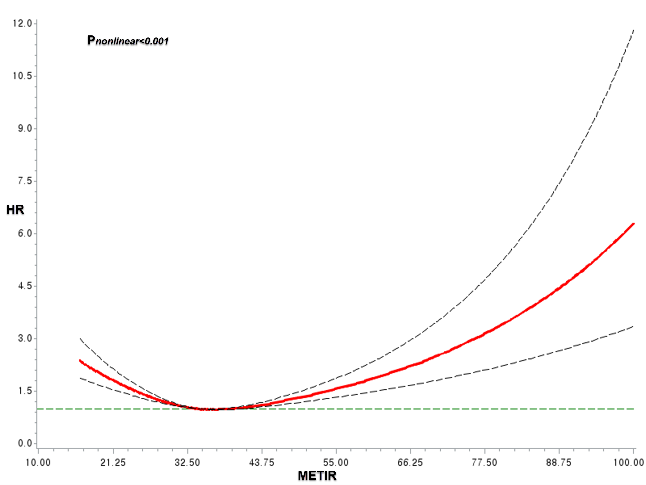 |
| 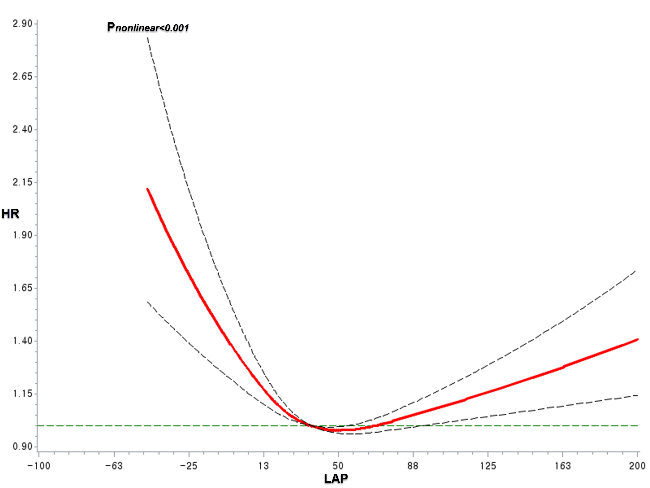 | 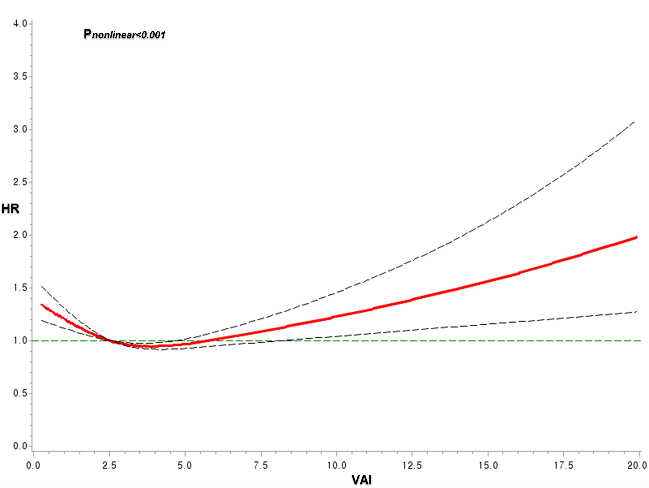 |

Fig.S6. **Nonlinear association between IR indices and all-cause mortality after excluding participants who died in the first 2 years of follow up. Events/total, 3,447/114,637.** The model was fitted using regression splines with three knots at the 5th, 50th, and 95th percentiles of IR indices. The red line indicates hazard ratios (HR), and the dotted lines indicate the 95% CI. The model was adjusted for age, sex, educational level, monthly income, marital status, smoking, drinking, regular physical exercise, and disease score. BMI, body mass index; TyG, triglyceride-glucose index; WC, waist circumference; VAI, visceral adiposity index; LAP, Lipid accumulation product; METS-IR, metabolic score for insulin resistance

| 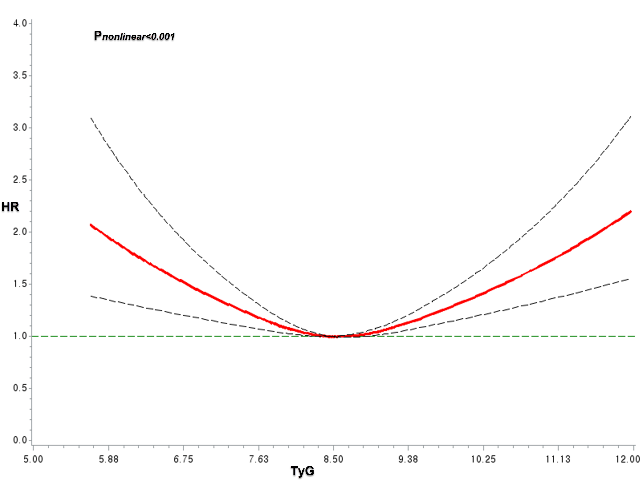 | 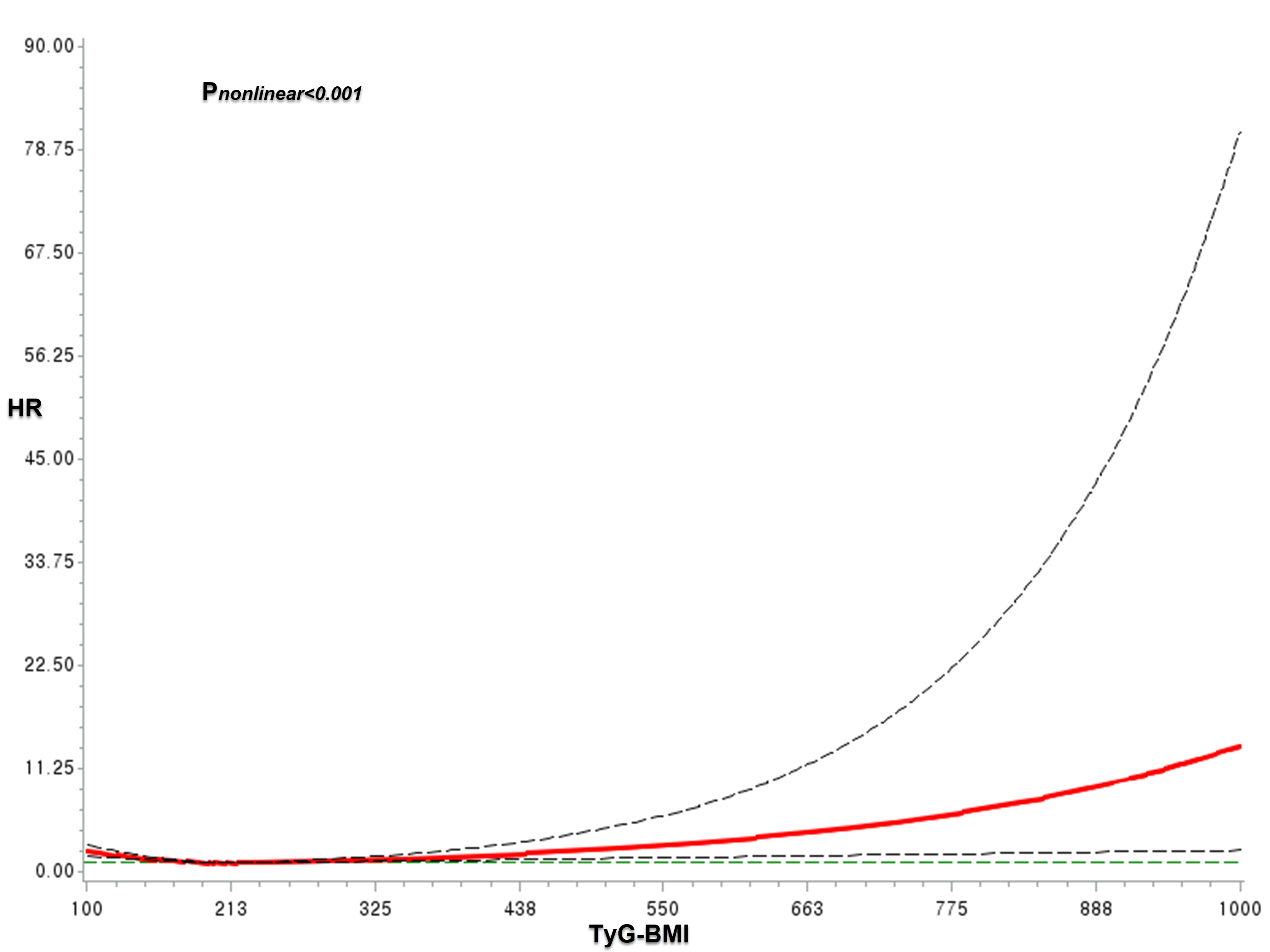 |
| --- | --- |
| 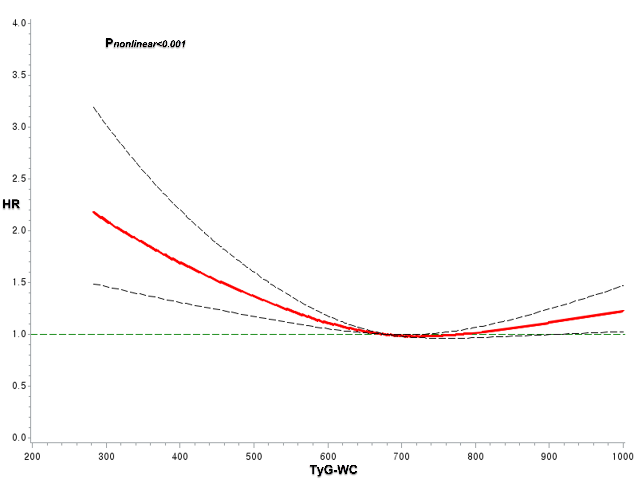 | 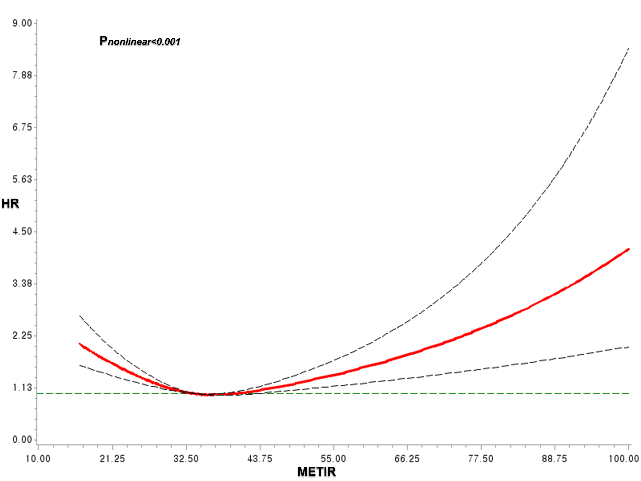 |
| 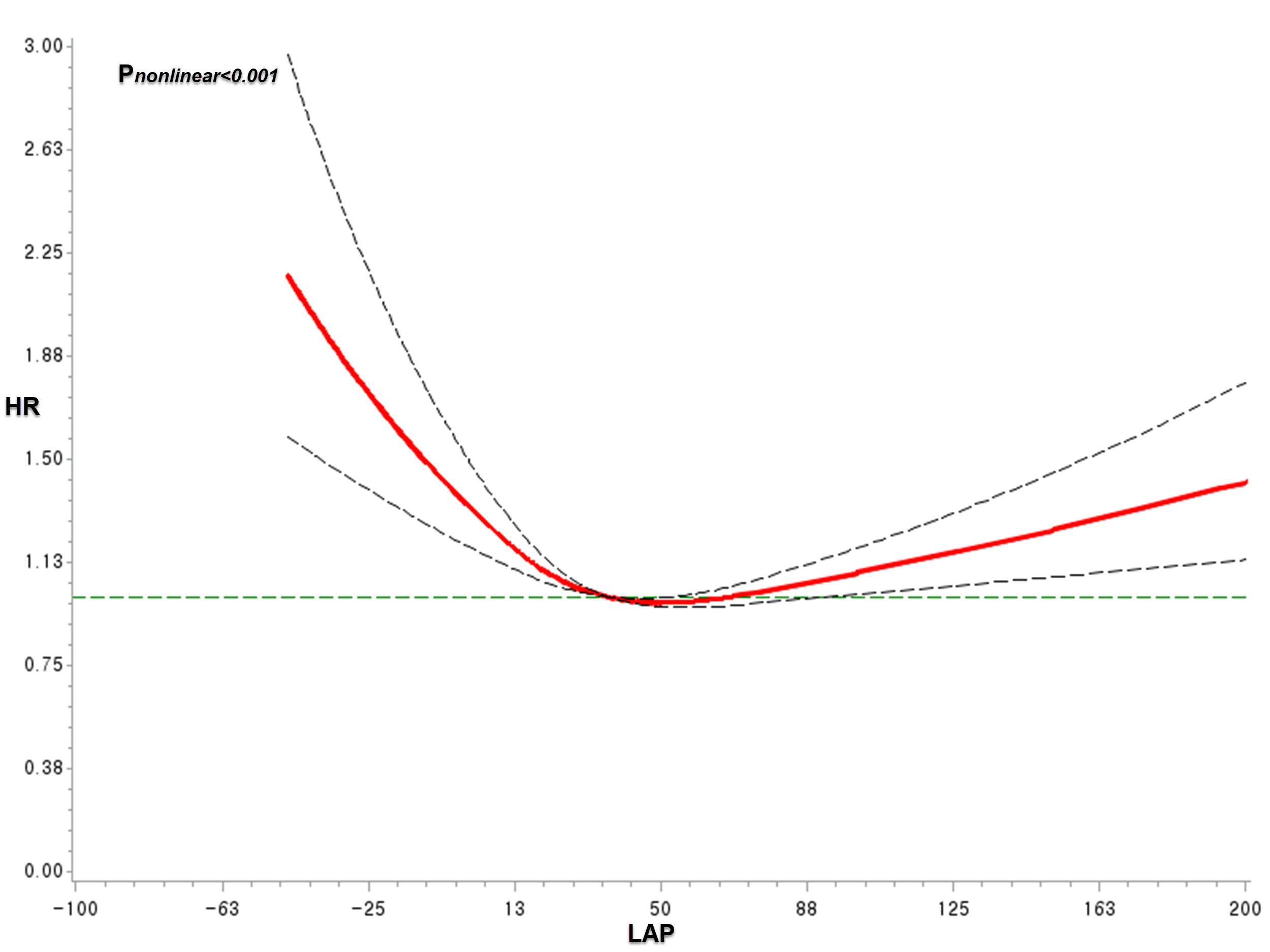 | 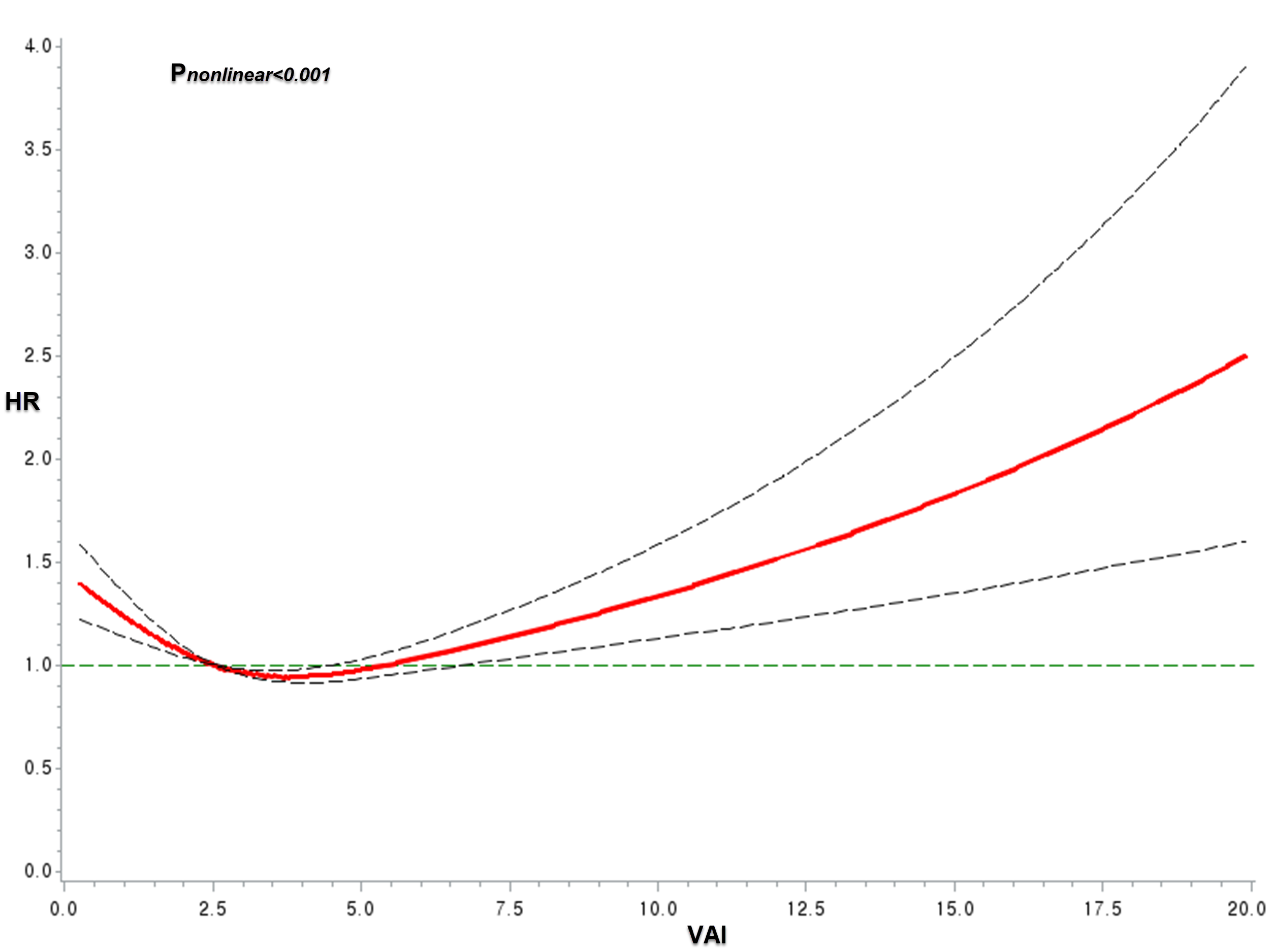 |

Fig.S7. **Nonlinear association between IR indices and all-cause mortality after excluding participants with a disease score>0 at baseline. Events/total, 2,914/105,260.** The model was fitted using regression splines with three knots at the 5th, 50th, and 95th percentiles of IR indices. The red line indicates hazard ratios (HR), and the dotted lines indicate the 95% CI. The model was adjusted for age, sex, educational level, monthly income, marital status, smoking, drinking, regular physical exercise. BMI, body mass index; TyG, triglyceride-glucose index; WC, waist circumference; VAI, visceral adiposity index; LAP, Lipid accumulation product; METS-IR, metabolic score for insulin resistance.

| 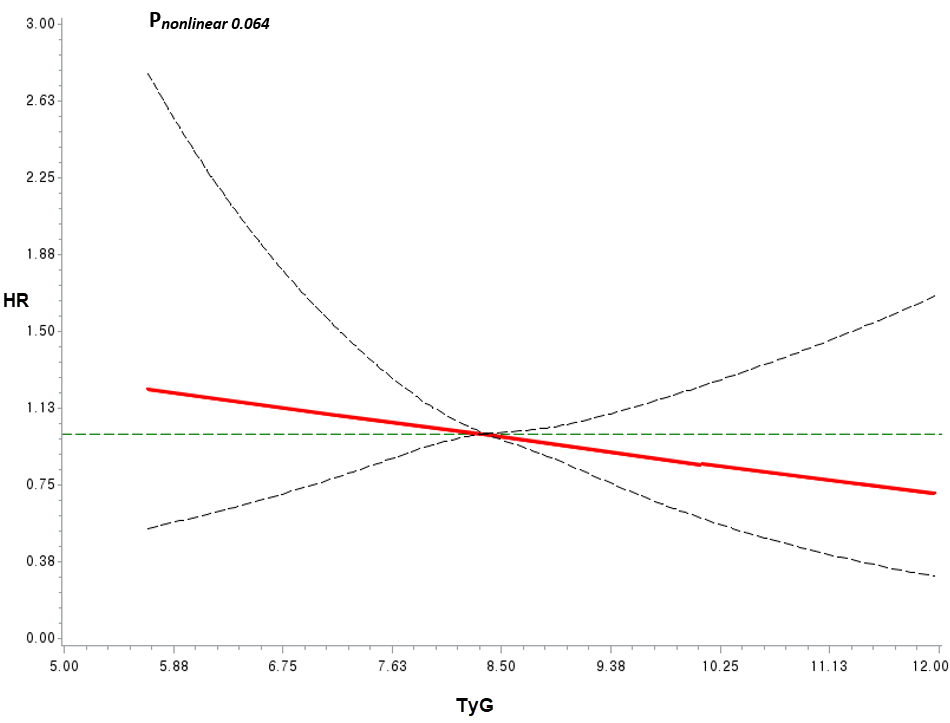 | 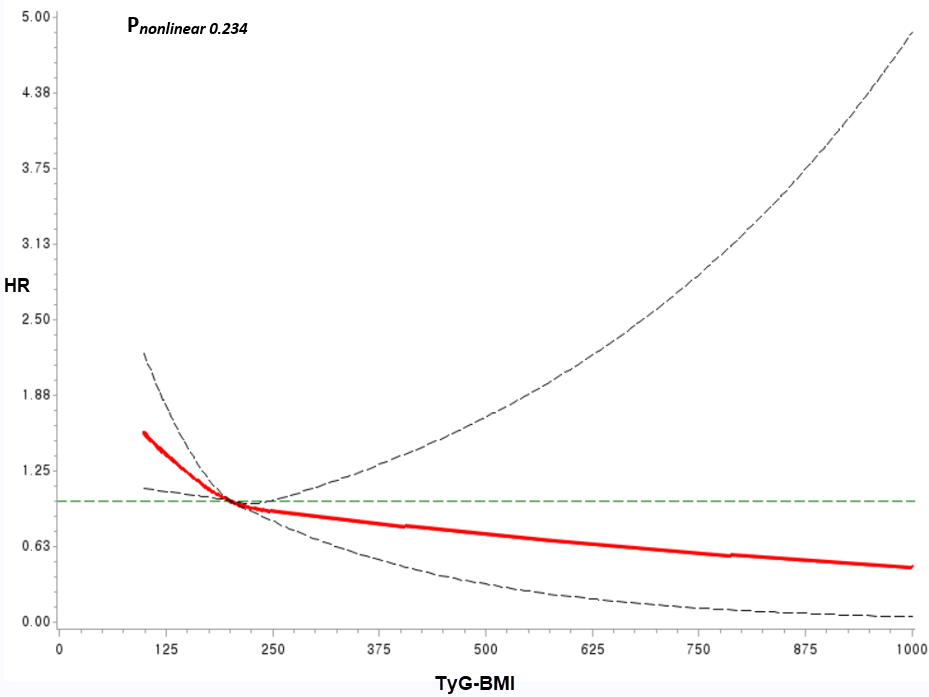 |
| --- | --- |
| 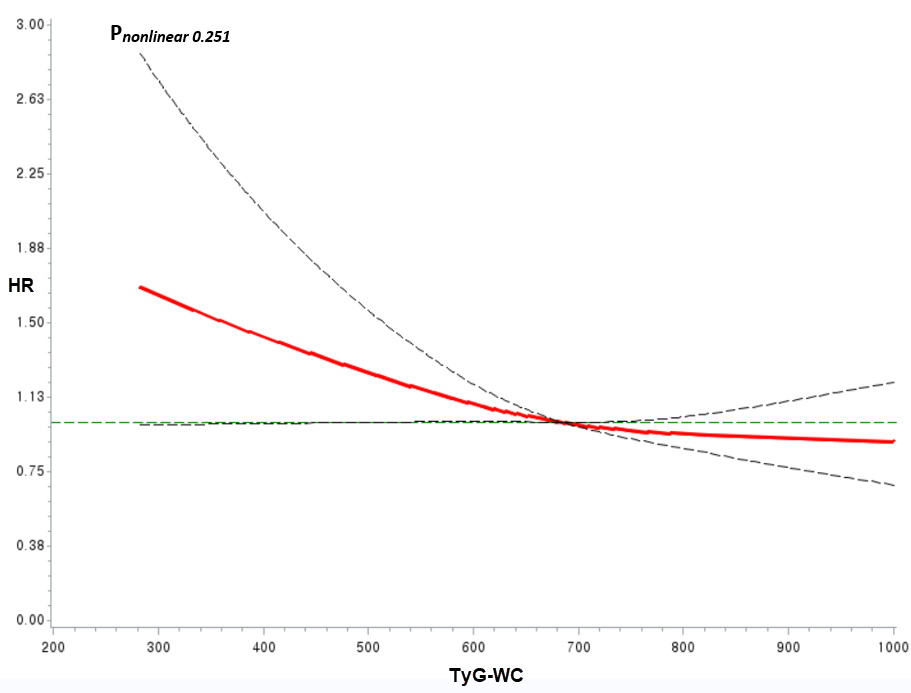 | 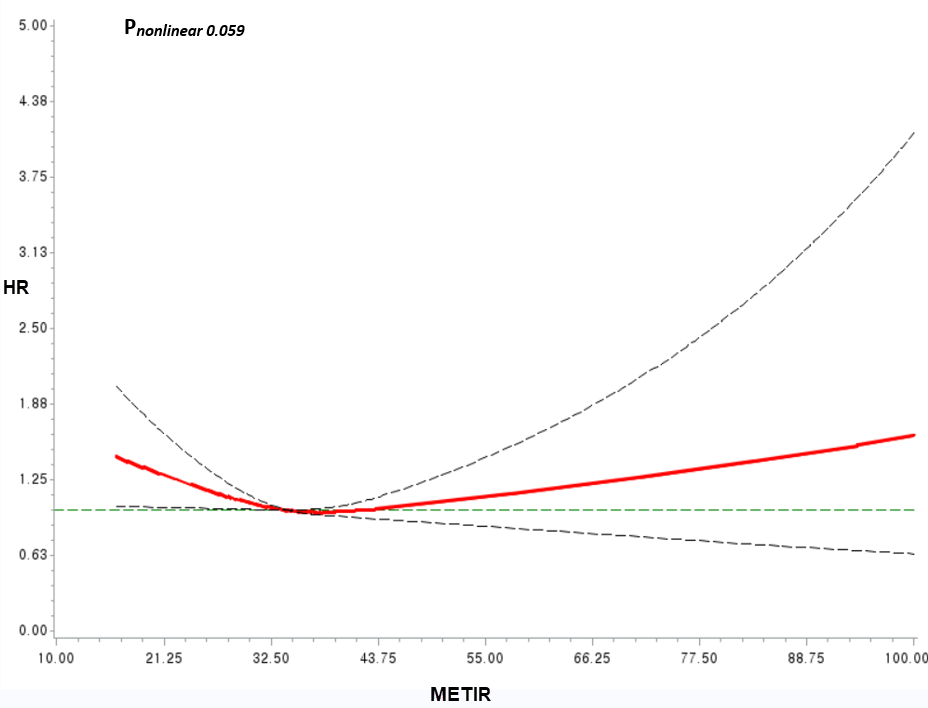 |
| 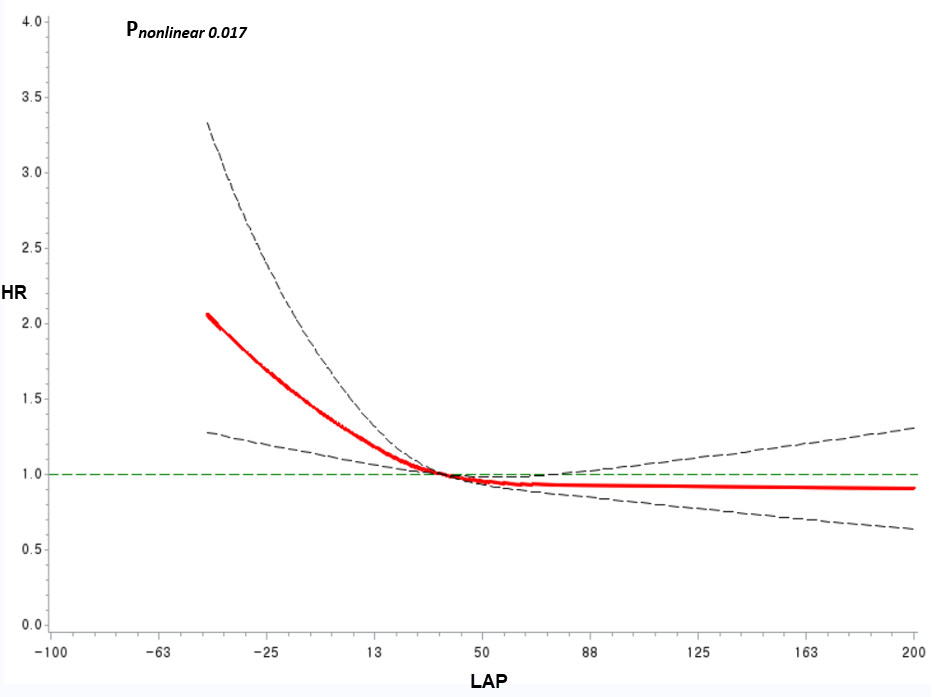 | 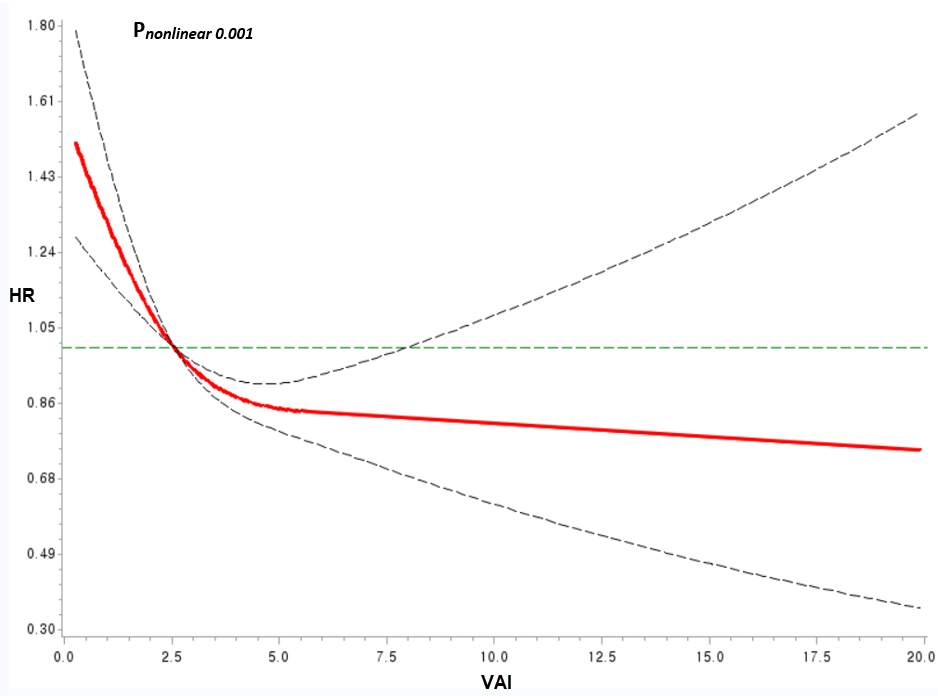 |

Fig.S9. Nonlinear association between IR indices and cancer mortality. The model was fitted using regression splines with three knots at the 5th, 50th, and 95th percentiles of IR indices. The red line indicates hazard ratios (HR), and the dotted lines indicate the 95% CI. The model was adjusted for age, sex, educational level, monthly income, marital status, smoking, drinking, regular physical exercise, and history of cancer. BMI, body mass index; TyG, triglyceride-glucose index; WC, waist circumference; VAI, visceral adiposity index; LAP, Lipid accumulation product; METS-IR, metabolic score for insulin resistance

| 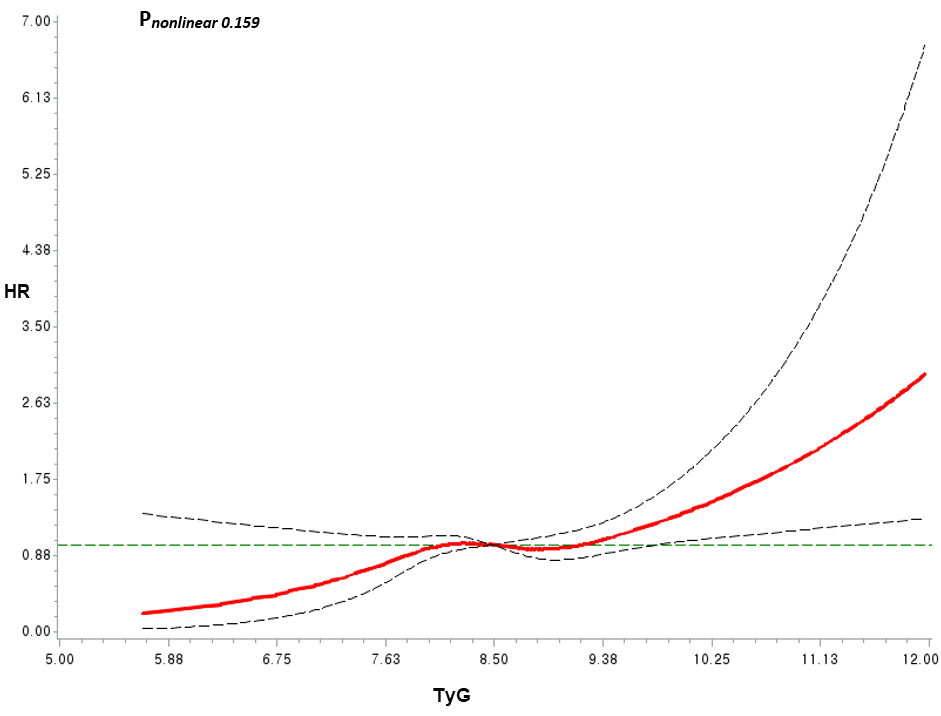 | 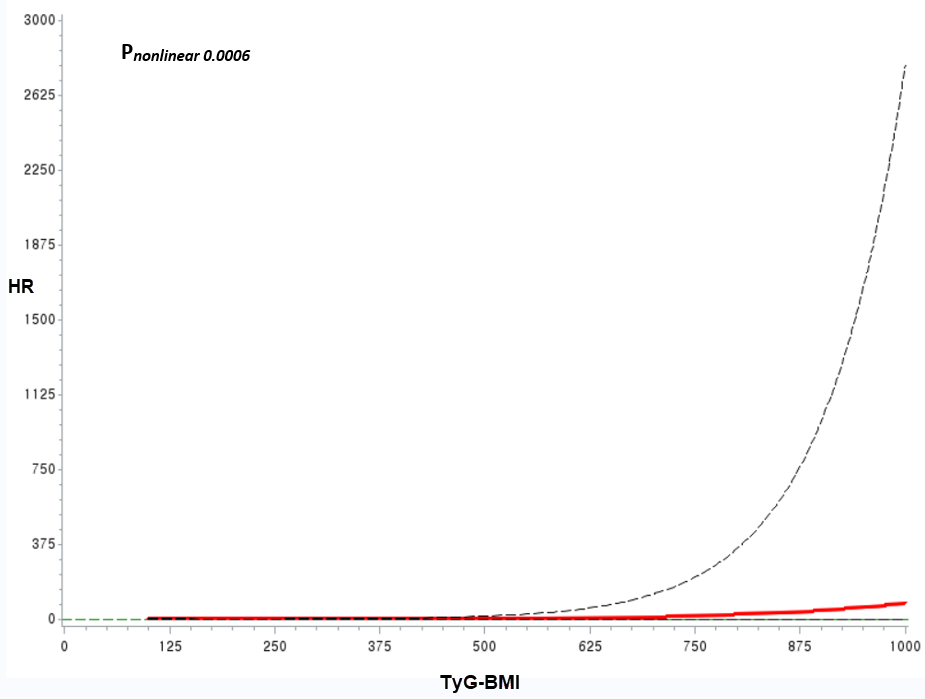 |
| --- | --- |
| 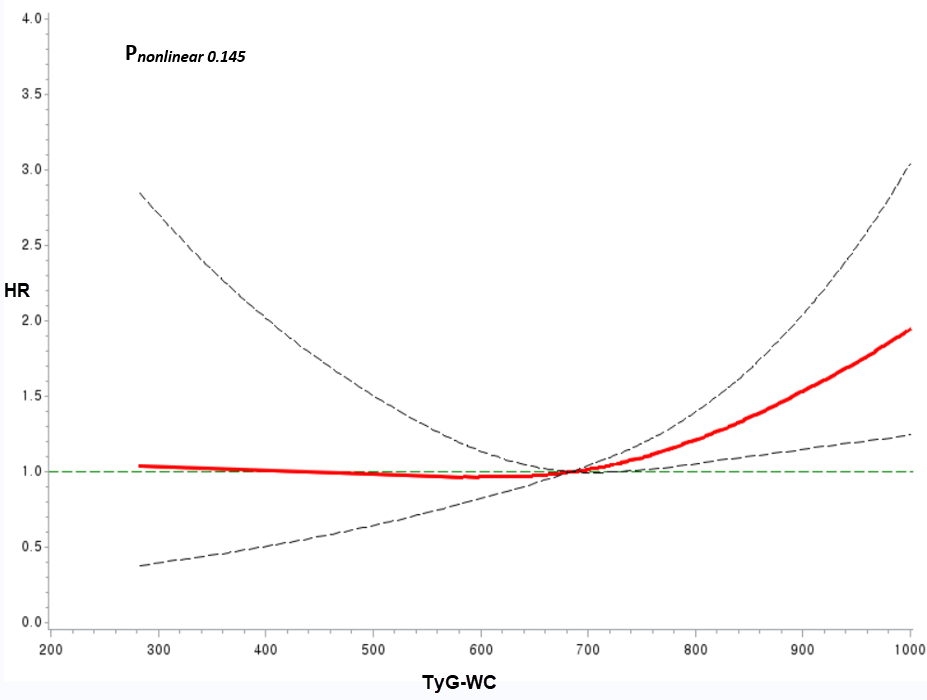 | 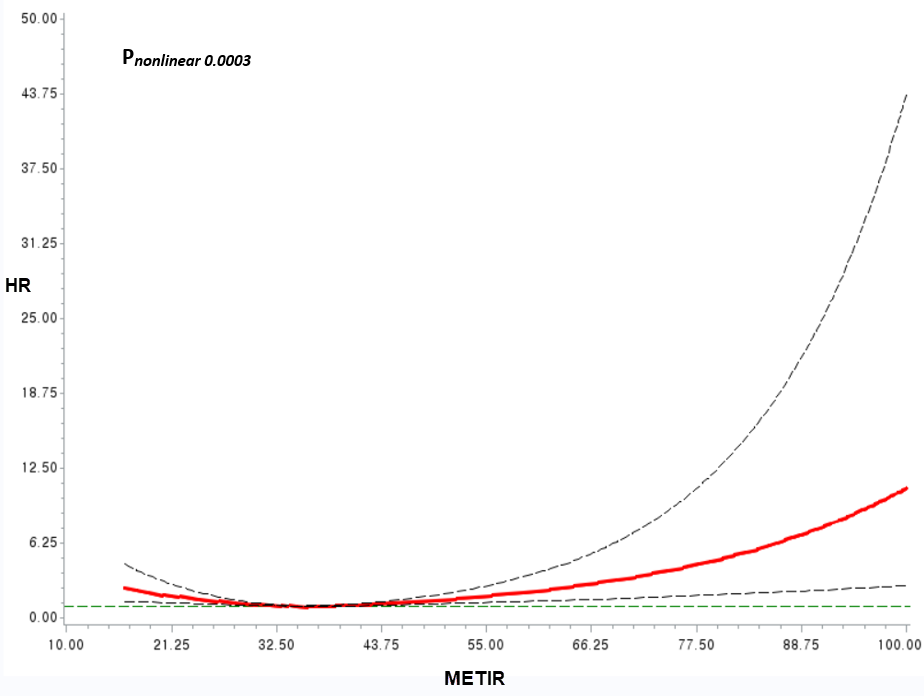 |
| 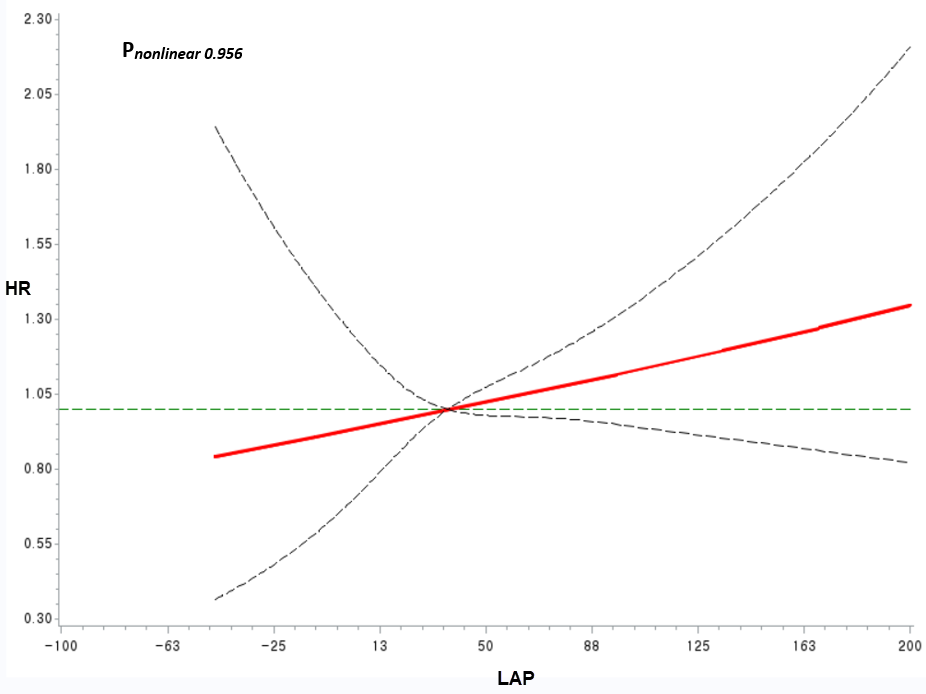 | 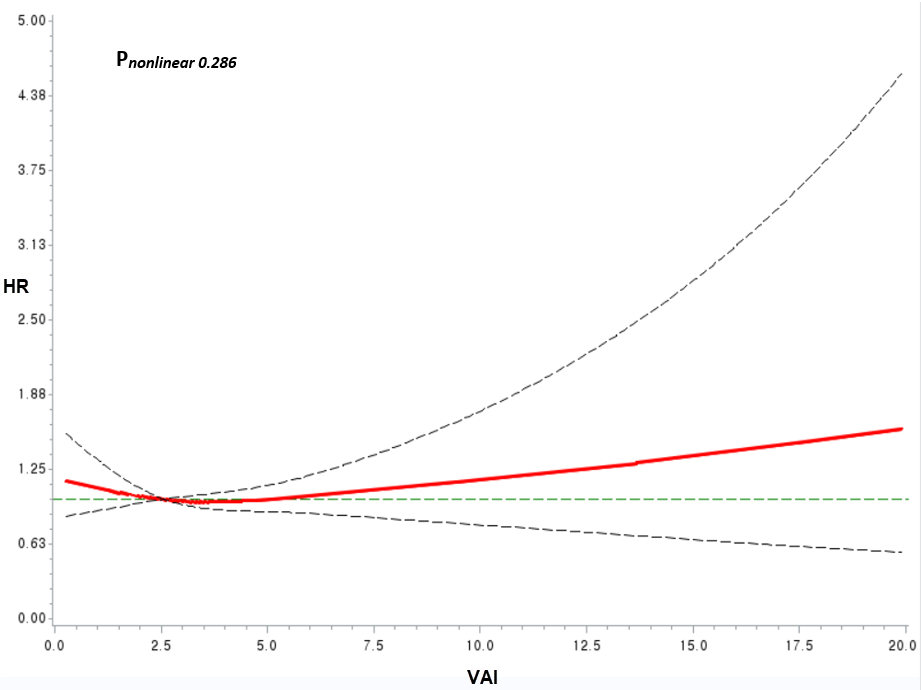 |

Fig.S10. Nonlinear association between IR indices and CVD mortality. The model was fitted using regression splines with three knots at the 5th, 50th, and 95th percentiles of IR indices. The red line indicates hazard ratios (HR), and the dotted lines indicate the 95% CI. The model was adjusted for age, sex, educational level, monthly income, marital status, smoking, drinking, regular physical exercise, and history of CVD. BMI, body mass index; TyG, triglyceride-glucose index; WC, waist circumference; VAI, visceral adiposity index; LAP, Lipid accumulation product; METS-IR, metabolic score for insulin resistance

Table S1. Characteristics of participants and non-participants.

| Characteristics |  | **Total** | **Non-participants** | **Participants** |
| --- | --- | --- | --- | --- |
| n |  | 173,195 | 58,238 | 114,957 |
| Age, years^a^ |  | 53.1 ± 0.03 | 53.0 ± 0.03 | 53.1 ± 0.03 |
| Sex, % | Men | 59,258 (34.2) | 19,552 (33.6) | 39,706 (34.5) |
|  | Women | 113,937 (65.8) | 38,686 (66.4) | 75,251 (65.5) |
|  |  |  |  |  |
| Education, % | Elementary | 30,262 (17.5) | 12,998 (22.3) | 17,264 (15.0) |
|  | Middle school | 277,96 (16.1) | 9,552 (16.4) | 18,244 (15.9) |
|  | High school | 68,137 (39.3) | 22,246 (38.2) | 45,891 (39.9) |
|  | College and above | 47,000 (27.1) | 13,442 (23.1) | 33,558 (29.2) |
|  |  |  |  |  |
| Income, % | Unknown | 29,131 (16.8) | 18,668 (32.1) | 10,463 (9.1) |
|  | < 1000 | 17,033 (9.8) | 5,822 (10.0) | 11,211 (9.8) |
|  | 1000-3000 | 62,433 (36.1) | 17,080 (29.3) | 45,353 (39.5) |
|  | ≥ 3000 | 64,598 (37.3) | 16,668 (28.6) | 47,930 (41.7) |
|  |  |  |  |  |
| Marital status, % | Single/others | 19,338 (11.2) | 7,277 (12.5) | 12,061 (10.5) |
|  | Married/cohabiting | 153,857 (88.8) | 50,961 (87.5) | 102,896 (89.5) |
|  |  |  |  |  |
| Residence, % | Seoul & Gyeongido | 67,787 (39.1) | 27,101 (46.5) | 40,686 (35.4) |
|  | Metropolitan city | 47,949 (27.7) | 10,277 (17.7) | 37,672 (32.8) |
|  | Others | 57,459 (33.2) | 20,860 (35.8) | 36,599 (31.8) |
|  |  |  |  |  |
| Smoking, % | Never | 126,650 (73.1) | 43,084 (74.0) | 83,566 (72.7) |
|  | Current | 25,205 (14.6) | 7,841 (13.5) | 17,364 (15.1) |
|  | Past | 21,340 (12.3) | 7,313 (12.6) | 14,027 (12.2) |
|  |  |  |  |  |
| Drinking, % | Never | 88,615 (51.2) | 29,712 (51.0) | 58,903 (51.2) |
|  | current | 77,672 (44.9) | 25,919 (44.5) | 51,753 (45.0) |
|  | Past | 6,908 (4.0) | 2,607 (4.5) | 4,301 (3.7) |
|  |  |  |  |  |
| Exercise, % | No | 112,540 (65.0) | 38,833 (66.7) | 73,707 (64.1) |
|  | Yes | 60,655 (35.0) | 19,405 (33.3) | 41,250 (35.9) |
| BMI, kg/m^2a^ |  | 23.9±0.01 | 24.0±0.01 | 23.9±0.01 |
| Triglycerides, mg/dL^a^ |  | 126.9±0.3 | 128.5±0.3 | 126.1±0.3 |
| Blood glucose, mg/dL^a^ |  | 95.2±0.1 | 95.2±0.1 | 95.1±0.1 |
| TyG^a^ |  | 8.5±0.00 | 8.5±0.00 | 8.5±0.00 |
| History of cancer, % | No | 167,926 (97.0) | 56,784 (97.5) | 111,142 (96.7) |
|  | Yes | 5,269 (3.0) | 1,454 (2.5) | 3815 (3.3) |
|  |  |  |  |  |
| History of CVD, % | No | 169,225 (97.7) | 56,995 (97.9) | 112,230 (97.6) |
|  | Yes | 3,970 (2.3) | 1,243 (2.1) | 2,727 (2.4) |
|  |  |  |  |  |
| Disease score, % | 0 | 157,652 (91) | 52,392 (90.0) | 105,260 (91.6) |
|  | ≥1 | 15,543 (9.0) | 5,846 (10.0) | 9,697 (8.4) |

^a^ mean±SE
